# Numerical Investigation of Gas–Liquid Multiphase Flow Characteristics and Nozzle Structure Optimization for a Multi-Hole Oxygen Lance in a Vanadium Extraction Converter

**DOI:** 10.3390/ma19163415

**Published:** 2026-08-12

**Authors:** Bowen Peng, Libin Yang, Jie Wang, Xiangchen Li, Chengyi Wang, Dengyu Niu, Congcong Zhang

**Affiliations:** 1 Central Iron & Steel Research Institute, Beijing 100081, China; 2 School of Metallurgy, Northeastern University, Shenyang 110819, China; 3 School of Metallurgical and Ecological Engineering, University of Science and Technology Beijing, Beijing 100083, China

**Keywords:** vanadium extraction converter, oxygen lance with a central nozzle, numerical simulation, jet coalescence, impact cavity morphology, molten bath mixing

## Abstract

**Highlights:**

The transient multiphase fluid dynamics and bath stirring mechanisms in a 200-t vanadium extraction converter were systematically quantified using a 3D CFD approach. * The aerodynamic interference and jet coalescence behaviors were critically compared between the central-hole (“3+1”) and conventional 3-hole nozzle configurations.

The mechanism by which inter-nozzle momentum allocation governs the morphological transition of the impact cavity (“deep and narrow” vs. “shallow and wide”) was elucidated.

An optimal 1:1 flow distribution ratio was identified, achieving the hydrodynamic synergistic optimization of lateral interface expansion and longitudinal deep-bath stirring.

**What are the main findings?**

For oxygen lances without a central nozzle: Lowering the design Mach number mitigates shock wave energy dissipation and enlarges the gas–liquid reaction surface area (up to 2.425 m^2^). However, due to the lack of central longitudinal penetrating force, a large stagnation dead zone is highly prone to forming at the bottom (volume fraction > 18.0%).

For oxygen lances with a central nozzle: The flow distribution dictates the flow field evolution. Excessive central flow causes the impact cavity to exhibit a “deep and narrow” profile and exacerbates surface kinetic energy dissipation, whereas insufficient central flow results in a “shallow and wide” cavity lacking adequate longitudinal momentum to drive deep circulation.

Regarding the optimal scheme: The “3+1” configuration adopting a 1:1 balanced flow ratio between the central nozzle and a single peripheral nozzle (Case 2#) is the optimal scheme. Compared to the prototype oxygen lance, this scheme reduces the jet coalescence deviation by 7.5%, increases the impact depth and reaction surface area by 29.8% and 17.0% (reaching 2.000 m^2^), respectively, and drastically minimizes the bottom inactive zone proportion to 0.14%.

**What are the implications of the main findings?**

Enhancing energy utilization efficiency: It is demonstrated that under equivalent oxygen consumption, optimizing the inter-nozzle momentum distribution can significantly improve the conversion efficiency of supersonic jet kinetic energy into macroscopic deep-bath circulation.

Guiding industrial practice: The optimized configuration and fluid dynamic laws obtained in this study provide highly valuable theoretical support and direct references for the engineering design, process parameter optimization, and subsequent industrial trials of novel oxygen lance nozzles in large-tonnage vanadium extraction converters.

**Abstract:**

To optimize the hydrodynamic conditions of the converter vanadium extraction process, a three-dimensional multiphase flow model of the top-blowing system in a 200 t vanadium extraction converter was established based on computational fluid dynamics (CFD). Conventional 3-hole and “3+1”-hole (with a central nozzle) oxygen lance schemes were designed to investigate the effects of nozzle design Mach numbers and inter-nozzle flow distributions on jet characteristics, impact cavity morphology, and internal molten bath flow fields, aiming to select the optimal oxygen lance nozzle structure. The results indicate that for oxygen lances without a central nozzle, lowering the design Mach number mitigates shock wave energy dissipation and enlarges the gas–liquid reaction surface area; however, due to the lack of longitudinal penetrating force from a central jet, a large stagnation dead zone is prone to forming at the bottom. For oxygen lances with a central nozzle, the inter-nozzle flow distribution governs the impact cavity morphology and the evolution of the flow field. Excessive central flow causes the impact cavity to exhibit a “deep and narrow” profile and exacerbates surface kinetic energy dissipation, whereas insufficient central flow results in a “shallow and wide” cavity that makes it difficult to drive deep circulation. Based on a multi-objective evaluation, the “3+1” configuration adopting a 1:1 balanced flow ratio between the central nozzle and a single peripheral nozzle (Case 2#) effectively balances the allocation of jet momentum between radial expansion and longitudinal penetration, achieving synergistic optimization of the gas–liquid reaction interface expansion and deep-bath stirring, thus serving as the optimal scheme. The findings of this study provide an important theoretical reference for the engineering design and industrial trials of oxygen lance nozzles in large-tonnage vanadium extraction converters.

## 1. Introduction

Within the comprehensive utilization framework of vanadium-titanium magnetite resources, the converter vanadium extraction process is characterized by operational conditions of short smelting cycles and low operating temperatures [1,2]. Given that the thermodynamic conditions for this selective oxidation are well established, further enhancement of the vanadium extraction efficiency relies primarily on improving the fluid dynamic conditions within the molten pool. This necessitates a synergy between the expansion of the gas–liquid interfacial area and the transmission of kinetic energy for deep stirring within the available smelting timeframe [3,4]. As the principal apparatus for oxygen supply and the provision of stirring kinetic energy to the molten pool, the nozzle geometric configuration of the top-blown oxygen lance (e.g., nozzle hole arrangement and design Mach number) dictates the aerodynamic evolution behavior of the multiple supersonic jets, which subsequently influences the impact morphology at the gas–liquid interface and the flow field distribution within the molten pool.

Systematic fundamental and applied research has been conducted by scholars worldwide concerning the aerodynamic characteristics of supersonic jets from oxygen lances. Regarding traditional oxygen lances without a central hole, previous studies [5,6,7,8] analyzed the gas flow characteristics within the Laval nozzle and the evolution behavior of the supersonic core region exterior to the nozzle; furthermore, Wang et al. [9], Wu [10], Zhang et al. [11], and Wang et al. [12] quantitatively investigated the comprehensive effects of parameters such as the design Mach number, nozzle inclination angle, ambient high-temperature fields [13], and oxygen supply intensity on jet attenuation; their studies demonstrated that optimizing nozzle design parameters can effectively mitigate the kinetic energy dissipation induced by wave structures. For the “3+1” type oxygen lance equipped with a central hole, the evolution of its flow field exhibits distinctly different characteristics. Studies by Yang et al. [14] and Feng et al. [15] indicated that the introduction of a central gas flow can generate a more uniform jet field; however, from the perspective of multi-jet interference, comparative studies by Pang et al. [16], Dong et al. [17], and Liu et al. [18] revealed that the central gas flow significantly intensifies the radially inward negative pressure gradient, triggering severe entrainment of ambient gas, which subsequently leads to exacerbated inward jet coalescence and momentum deflection.

In terms of the jet-bath interaction between the top-blown jet and the molten pool, different nozzle configurations lead to distinct dynamic evolution characteristics within the bath. For traditional oxygen lances without a central hole, Wang et al. [9] and Wang et al. [19], based on numerical simulations of gas-slag-metal multiphase flow, evaluated the effects of nozzle inclination angle, lance height, and design Mach number on the residual kinetic energy reaching the liquid surface. The results demonstrated that adjusting the aforementioned parameters can alter the shear stress at the gas–liquid interface and the impact cavity morphology. However, regarding the molten pool of large-scale vanadium extraction converters, kinetic studies by Zhen [20], Zhou [21], and Zhang [22] indicated that owing to the weak decarburization reaction during the vanadium extraction period, the interior of the molten pool lacks the self-stirring effect generated by the floating of massive CO bubbles. Under such operating conditions, the homogenization of bath composition and temperature relies on the mechanical stirring of the top-blown jet; the lateral impact associated with traditional configurations is prone to causing insufficient kinetic energy in deep fluids, resulting in the formation of an extensive low-velocity stagnation region (dead zone) at the bottom [3]. Studies by Liu et al. [18] on the structure equipped with a central hole demonstrated that the central jet deepens the local impact cavity and promotes the upward recirculation of deep fluids. However, the inward jet coalescence phenomenon compromises the morphological stability of the impact cavity edge, leading to a reduction in the lateral gas–liquid reaction area [16]. This indicates that both configurations need to seek a reasonable dynamic balance between expanding the lateral gas–liquid reaction interface and enhancing the longitudinal deep kinetic energy.

In summary, despite extensive research in the existing literature on oxygen lance jets and gas–liquid coupling behaviors, the adaptability and optimization strategies concerning the geometric parameters of the oxygen lance nozzle and the multiphase flow evolution within the molten pool of a 200-t converter, under the specific oxygen supply parameters of the vanadium extraction process, remain to be further refined. Regarding the “3+1” structure equipped with a central hole, there is a lack of specific research on how the flow distribution ratio between the central and peripheral holes alters the degree of interference among multiple jets, thereby determining the longitudinal penetration depth and lateral extension range at the gas–liquid interface. Concurrently, under an equivalent oxygen supply flow rate for vanadium extraction, the capacity of the traditional three-hole oxygen lance to expand the gas–liquid reaction interface by adjusting the design Mach number, alongside the specific differences in the bath stirring effect between this configuration and the central-hole structure, necessitates further comparative analysis.

To this end, targeting the multi-hole oxygen lance of a 200-t vanadium extraction converter, this study designed nozzle configurations of a traditional type without a central hole (3 holes) and a type equipped with a central hole (1 central nozzle + 3 peripheral nozzles), and subsequently established a three-dimensional numerical model of gas–liquid multiphase flow for the top-blown converter. Through numerical simulations, the impacts of the design Mach number and the central hole flow distribution ratio on the dynamic characteristics of multiple jets, the morphological evolution of the impact cavity, and the flow field within the molten pool were analyzed, thereby providing an engineering optimization basis for the oxygen supply process of large-tonnage vanadium extraction converters.

## 2. Mathematical Model

### 2.1. Governing Equations

#### 2.1.1. Model Assumptions

In accordance with the actual operating conditions, the following assumptions were made to establish the numerical model of multiphase flow within the 200-t vanadium extraction converter:

(1) Oxygen is treated as a compressible ideal gas.
(2) The molten steel is regarded as an incompressible and isothermal Newtonian fluid.
(3) No mass transfer or chemical reactions occur between the gas and liquid phases; only momentum and energy exchanges are considered.
(4) Both the supersonic jet and the multi-phase molten pool flow are resolved using a transient (time-dependent) numerical approach. The Standard k−ε turbulence model is applied to resolve the time-averaged fluid quantities, while the Volume of Fluid (VOF) model precisely tracks the transient spatial fluctuations of the gas–liquid interface. The mathematical analysis within the 1.0–2.0 s computational window represents a quasi-steady oscillatory state, and all reported spatial flow field properties are extracted utilizing a time-averaging methodology.
(5) All wall boundaries are assumed to be smooth, with frictional resistance neglected.

#### 2.1.2. Justification of Metallurgical Simplifications

The physical simplifications adopted in this numerical model are intrinsically justified by the distinct thermodynamic and kinetic characteristics of the converter vanadium extraction process. Unlike conventional steelmaking, this specific metallurgical stage operates under relatively low-temperature conditions (approximately 1623 K) to facilitate the selective oxidation of vanadium while strictly suppressing carbon oxidation. Consequently, the extremely weak decarburization reaction dictates that the massive evolution of CO gas bubbles—and its associated buoyancy-driven self-stirring effect—is virtually non-existent within the bath. This physical reality renders the assumption of a non-reacting, isothermal molten pool mathematically and physically appropriate, as the macroscopic deep-bath circulation is overwhelmingly driven by the mechanical momentum transferred from the top-blown supersonic jets. Furthermore, the exclusion of the top slag phase serves to prevent severe numerical divergence of the VOF interface under extreme supersonic shear stresses. Given the massive spatial scale of the 200 t converter, the momentum dissipation induced by boundary friction at the smooth walls represents a negligible energy sink compared to the intense axial kinetic energy imparted by the multi-hole jets. Thus, treating the liquid steel as a continuous, isothermal Newtonian fluid isolates and highlights the core hydrodynamic efficiency of the different oxygen lance nozzle configurations.

#### 2.1.3. Governing Equations

Based on the aforementioned assumptions, the conservation equations for mass, momentum, and energy are expressed as follows:

(1) Mass conservation equation:

(1) $$\frac{\partial \rho }{\partial t}+\nabla \cdot (\rho U)=0$$

where ρ is the average fluid density, kg/m^3^; t is the time, s; and U is the jet velocity, m/s.

(2) Momentum conservation equation:

(2) $$\begin{array}{c} \frac{\partial }{\partial t}(\rho U)+\nabla \cdot (\rho U\times U)-\nabla \cdot (\mu_{\mathrm{eff}}\nabla U)\\ =-\nabla P+\nabla [\mu_{\mathrm{eff}}(\nabla U)]+F \end{array}$$

where P is the static pressure (MPa), F is the external body force (N), and τeff is the effective viscosity (Pa·s).

(3) Energy conservation equation:

(3) $$\frac{\partial }{\partial t}(\rho H)+\nabla \cdot (\rho UH)-\nabla \cdot (\lambda \nabla T)=\frac{\partial P}{\partial t}$$

where *λ* is the thermal conductivity (W/(m·K)), *T* is the temperature (K), and *H* is the total enthalpy (kJ/mol).

#### 2.1.4. Turbulence Model Validation and VOF Model Volume Fraction Equation

In the smelting environment of a full-scale converter, the high Reynolds number characteristics of the multi-hole supersonic oxygen jets and their gas–liquid interactions with the molten pool constitute the key factors influencing macroscopic hydrodynamic behaviors. Recent literature has employed the Standard k-ε model [23] in conjunction with the VOF method [7,24] to successfully predict the core aerodynamic attenuation behavior of supersonic jets from Laval nozzles, as well as the multiphase stirring behaviors within industrial-scale converters. Taking into comprehensive consideration the massive three-dimensional spatial scale of the 200-t vanadium extraction converter, alongside the necessity to ensure computational convergence in the high Reynolds number free-stream regions of the multi-hole jets, this study also selected this model to predict the supersonic jet behaviors and the gas–liquid dynamic responses of the molten pool; the transport equations for the turbulent kinetic energy and the turbulent dissipation rate are formulated as follows:

Equation for the turbulent kinetic energy *k*:

(4) $$\rho \frac{\partial (v_{j}k)}{\partial x_{j}}=\frac{\partial }{\partial x_{j}}\left(\frac{\mu_{t}}{\sigma_{k}}\frac{\partial k}{\partial x_{j}}\right)+G-\rho \varepsilon +S_{k}$$

Equation for the turbulent dissipation rate *ε*:

(5) $$\rho \frac{\partial (v_{j}\varepsilon )}{\partial x_{i}}=\frac{\partial }{\partial x_{i}}\left(\frac{\mu_{t}}{\sigma_{\varepsilon }}\frac{\partial \varepsilon }{\partial x_{j}}\right)+\frac{\left(C_{1}G_{\varepsilon }-C_{2}\rho \varepsilon^{2}\right)}{k}+S_{\varepsilon }$$

where $G=\mu_{t}\frac{\partial v_{j}}{\partial x_{i}}\left(\frac{\partial v_{i}}{\partial x_{j}}+\frac{\partial v_{j}}{\partial x_{i}}\right)$, where $\mu_{t}$ is the turbulent viscosity coefficient; the empirical constants for the turbulence models are $C_{1}$ = 1.44, $C_{2}$ = 1.92, $\sigma_{k}$ = 1.0, and $\sigma_{\varepsilon }$ = 1.3; and $S_{\varepsilon }$ is the source term (kg/(m·s^4^)).

Volume fraction equation of the VOF model, where the gas phase volume fraction is *α*, and the sum of the volume fractions of gas and liquid in each cell equals 1. There are three conditions within the computational domain:

α = 1: the cell is completely filled with gas;

α = 0: the cell is completely filled with liquid;

0 < α < 1: the cell contains both gas and liquid, indicating the presence of a gas–liquid interface.

The continuity equation for the VOF model is as follows:

(6) $$\frac{1}{\rho_{g}}\left[\frac{\partial }{\partial t}\left(\alpha_{g}\rho_{g}\right)+\nabla \cdot \left(\alpha_{g}\rho_{g}v_{g}\right)\right]=S_{\alpha g}+\sum_{l=1}^{2}\left(m_{lg}-m_{gl}\right)$$

where $m_{gl}$ is the mass transferred from the gas phase to the liquid phase, and $m_{lg}$ is the mass transferred from the liquid phase to the gas phase.

### 2.2. Model Establishment and Boundary Conditions

**Figure 1:**
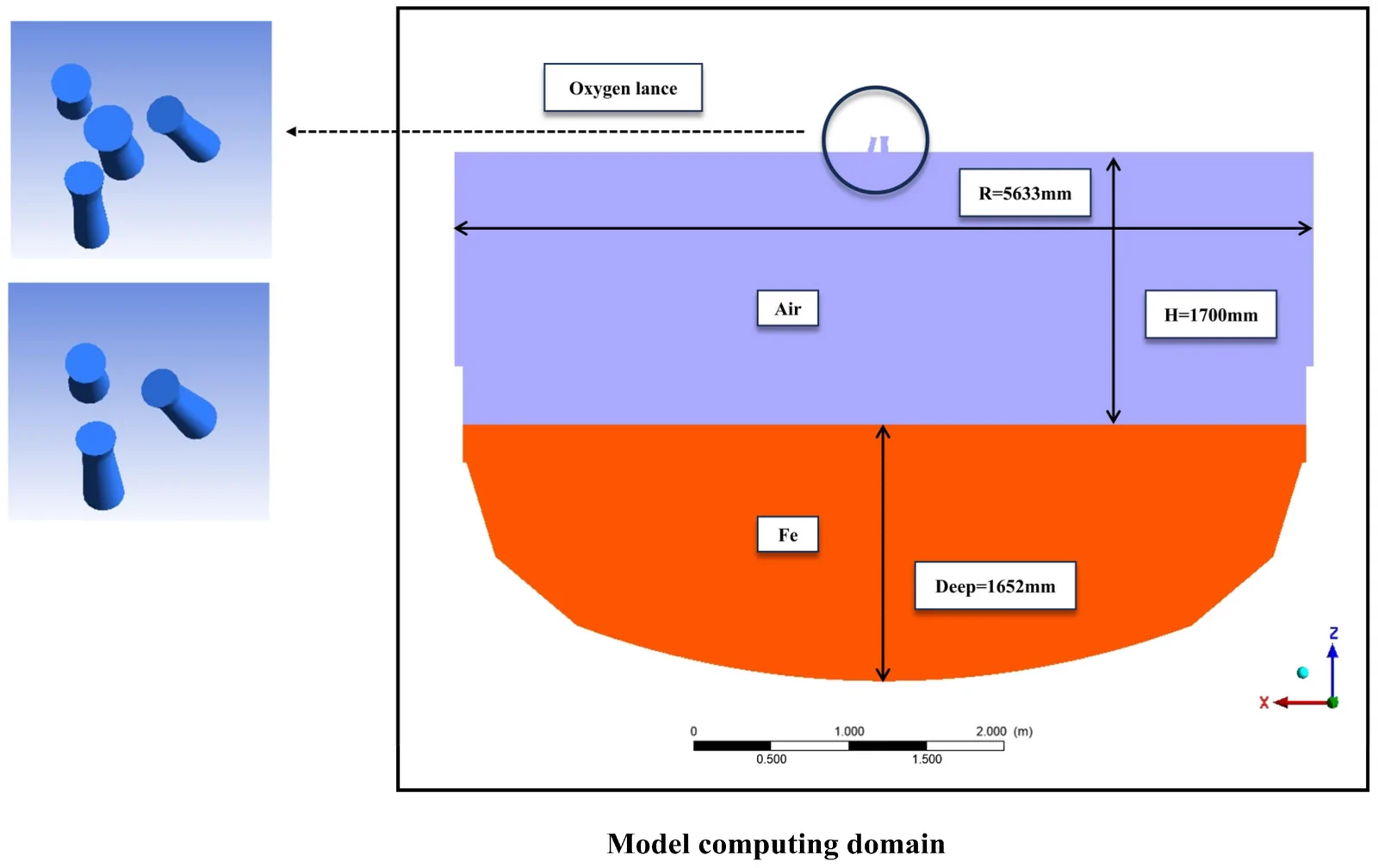
Geometric models of different oxygen lances and schematic diagrams of converter longitudinal sections.

**Figure 2:**
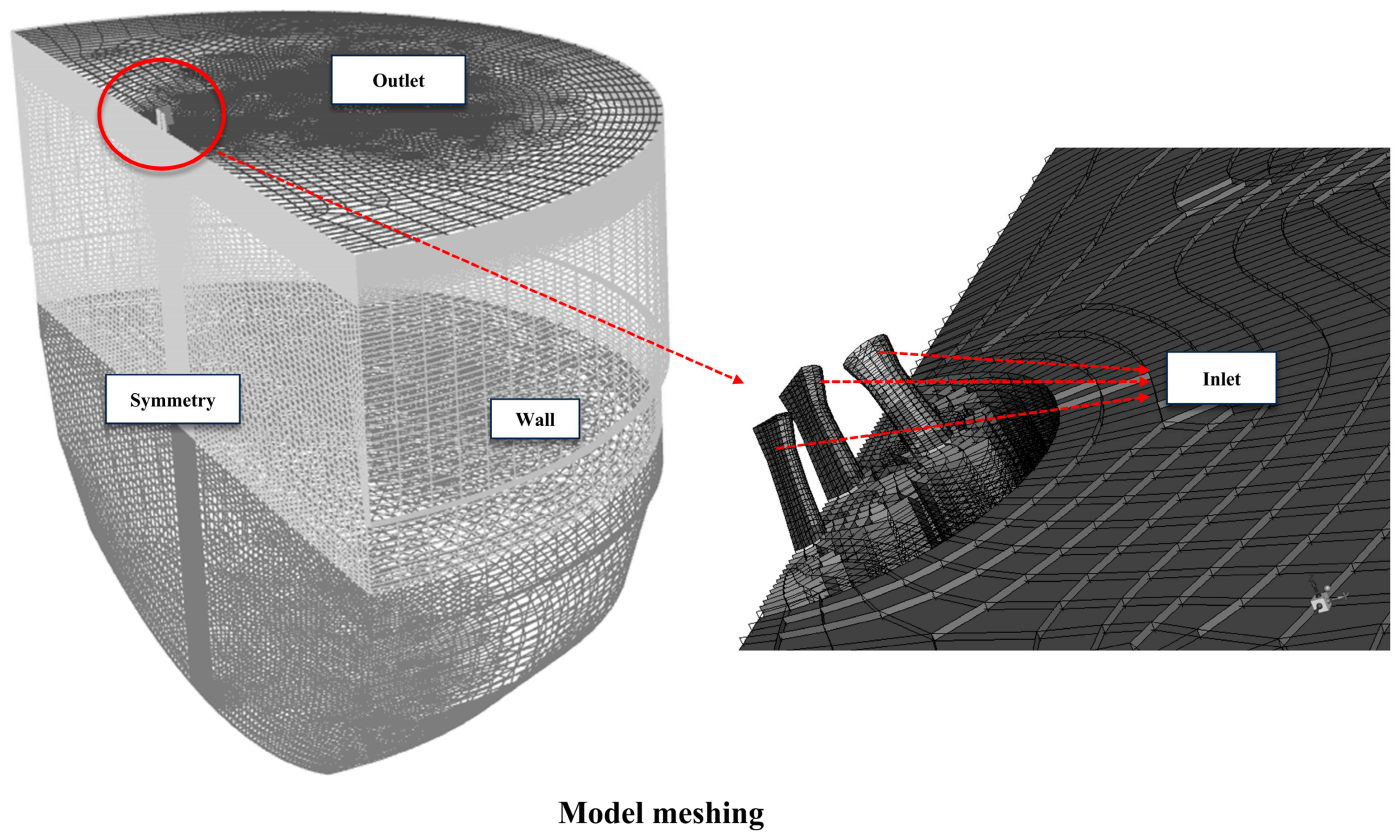
Grid model illustration.

Using scheme No. 1 (comprising one central nozzle and three peripheral nozzles) from the 200-t vanadium extraction converter field application as the benchmark, seven different lance schemes were designed and compared by varying the nozzle throat and exit diameters; the model parameters are depicted in Figure 1. Among these, schemes No. 2, 3, and 4 belong to the “3+1” hole structures with a central nozzle to investigate the influence of flow distribution, wherein the design exit Mach numbers for both the central and peripheral nozzles are maintained at 2.06, and the flow ratios of the central hole to a single peripheral hole are set at 1:1, 2:1, and 1:1.5, respectively; schemes No. 5 to 7 represent the traditional 3-hole structures without a central nozzle. A 1/2 computational domain was constructed based on the physical symmetry of the model to balance computational accuracy and efficiency. The structural parameters of the oxygen lance nozzles are listed in Table 1, the operating boundary conditions are provided in Table 2, and the mesh model is shown in Figure 2.

In this study, the standard model proposed by Launder and Spalding [23] was adopted to determine the turbulent kinetic energy and its dissipation rate. The commercial software Fluent 2021 was utilized to solve the turbulence and Volume of Fluid (VOF) multiphase flow models. The computational time step was set to $1.0\times 10^{-5}$ s. With the exception of the residual convergence criterion for the energy equation, which was set to $1.0\times 10^{-6}$, the root-mean-square (RMS) residual convergence criteria for all other physical quantities were uniformly set to $1.0\times 10^{-3}$.

### 2.3. Model Validation

#### 2.3.1. Mesh Independence Verification

**Figure 3:**
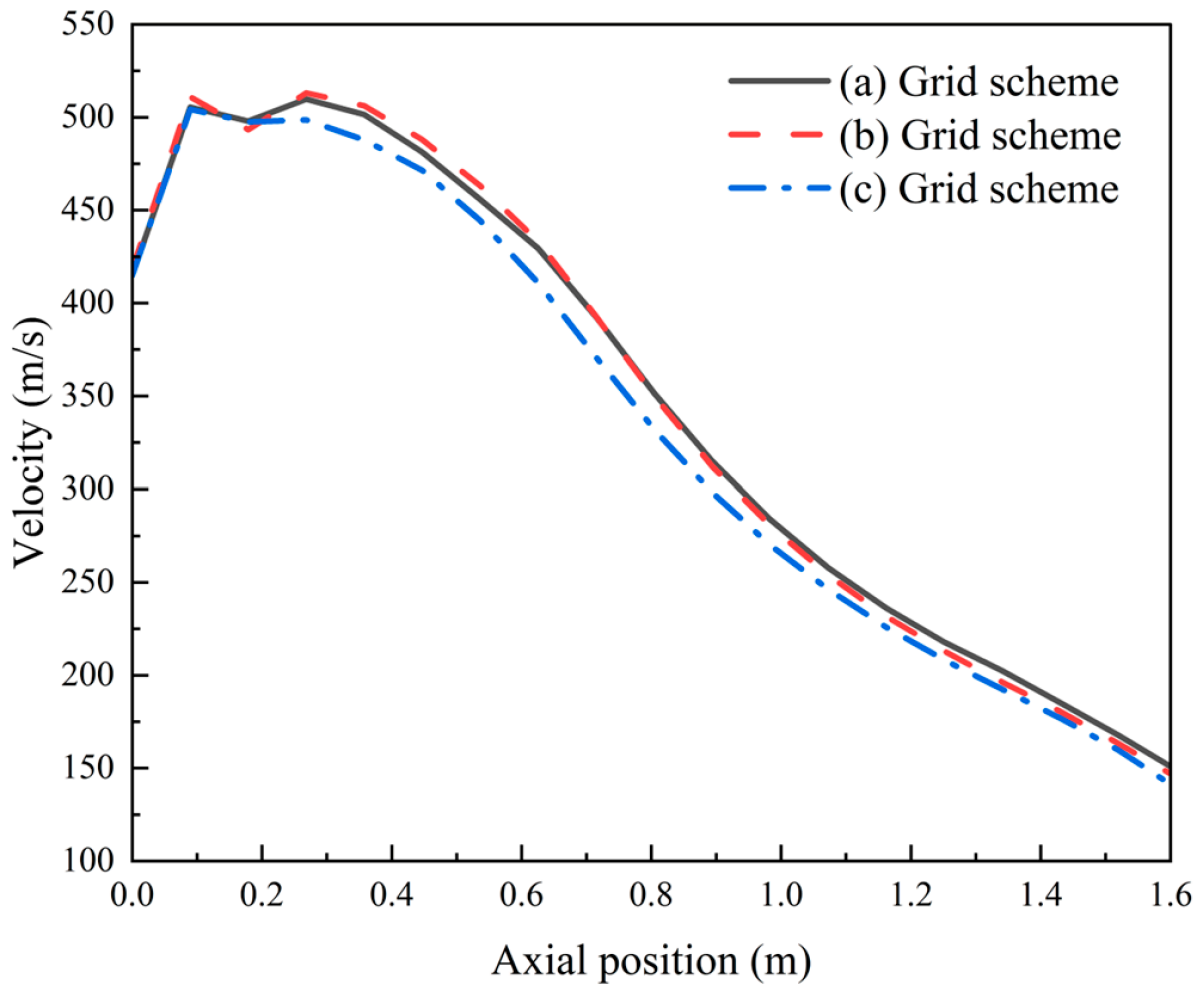
Central nozzle jet at different grid schemes.

Mesh independence verification was conducted using a three-level hexahedral mesh refinement strategy: a base mesh (Scheme a) with approximately 3.0 × 10^5^ cells, a medium mesh (Scheme b) with approximately 5.0 × 10^5^ cells, and a fine mesh (Scheme c) with approximately 7.0 × 10^5^ cells. Taking oxygen lance Scheme 1# as the benchmark, Figure 3 illustrates the axial velocity distribution along the centerline of the supersonic jet under different mesh schemes. To rigorously quantify the spatial discretization error, the Grid Convergence Index (GCI) based on Roache’s methodology was implemented. The time-averaged axial jet velocity at Z = 0.5 m along the centerline of the 1# oxygen lance was selected as the critical monitoring variable. Utilizing the three mesh levels—base (3.0 × 10^5^), medium (5.0 × 10^5^), and fine (7.0 × 10^5^)—the velocity outputs exhibited a typical oscillatory convergence behavior. According to the ASME V&V 20 guidelines for oscillatory convergence, the theoretical order of accuracy (*p* = 2 for the second-order upwind spatial discretization scheme) was adopted for the calculation. The numerical analysis revealed that the relative error between the medium and fine meshes is merely 0.66%, and the calculated $GCI_{medium,fine}$ value is 3.27%. This value is strictly below the conservative threshold of 5%. This mathematical verification confirms that the spatial discretization error of the medium mesh (Scheme b) is negligible, thereby establishing absolute numerical independence. The detailed GCI parameters are listed in Table 3. Considering both numerical accuracy and computational efficiency, the medium mesh (Scheme b) was selected for all subsequent optimization simulations.

#### 2.3.2. Time Discretization and Numerical Stability

For transient supersonic jets with peak velocities exceeding 500 m/s, time discretization must strictly adhere to the Courant-Friedrichs-Lewy (CFL) criterion. The computational time step was set to Δt = 2.0 × 10^−5^ s. This temporal resolution ensures that the local CFL number throughout the entire computational domain remains below 1.0, effectively preventing numerical divergence of the VOF model while tracking complex gas–liquid impact interfaces and transient fluctuations.

#### 2.3.3. Model Validation

To ensure that the numerical model can accurately predict the jet behavior within the 200-t vanadium extraction converter, the computational model for the 1# oxygen lance (“3+1” hole structure) was validated from two dimensions: the initial thermodynamic boundaries and the spatial evolution characteristics of the flow field. First, the CFD simulation values of the aerodynamic parameters at the central nozzle exit were compared with the theoretical values derived from one-dimensional steady isentropic expansion (see Table 4). Given that a 1/2 symmetric model was employed for the computational domain, the integrated mass flow rate at the inlet section of the central hole (1.669 kg/s) is equivalent to a full-nozzle physical flow rate of 3.34 kg/s. The results indicate that the relative errors for the exit Mach number and mass flow rate are 3.97% and 2.10%, respectively, both of which satisfy engineering accuracy requirements; this confirms the accuracy of the model in describing supersonic compressible flow and mass conservation characteristics within the Laval nozzle.

**Figure 4:**
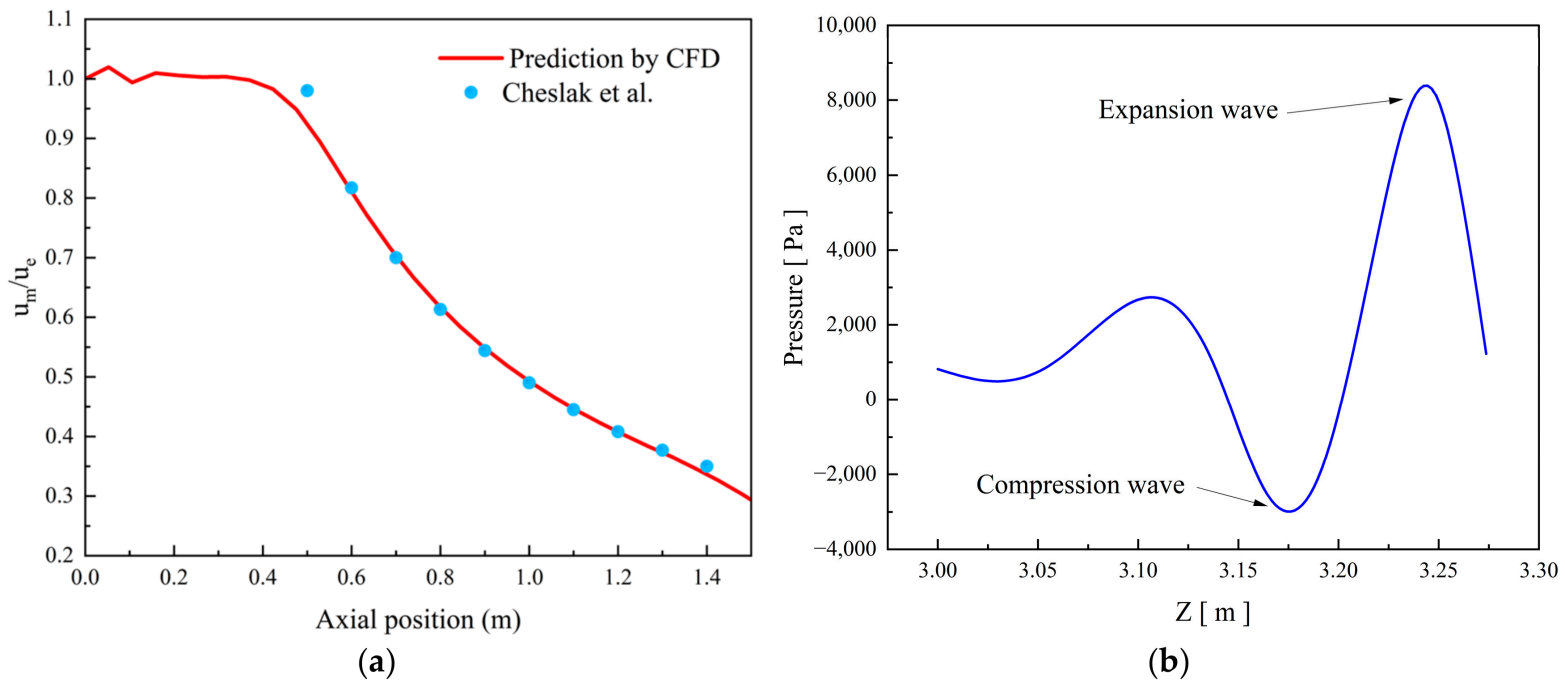
Numerical model validation: (**a**) Axial dimensionless velocity distribution compared with Cheslak’s semi-empirical formula; (**b**) Static pressure distribution along the central jet axis illustrating the shock-wave characteristics [25].

Furthermore, dimensionless velocity data along the centerline of the central hole jet were extracted and subjected to non-linear fitting with Cheslak’s semi-empirical formula [25] to verify the evolution characteristics of the spatial flow field. The fitting results (shown in Figure 4) reveal that the axial velocity decay proportionality constant for the specific “3+1” structure in this study is 8.67. This value is higher than the empirical range for traditional single-hole jets (5.13–7.7), which is attributed to the fact that, compared to a single-hole jet, the peripheral nozzle jets provide aerodynamic shielding for the central jet; this effectively diminishes the shearing and entrainment effects of the ambient gas on the main jet, thereby delaying momentum dissipation in the core region. The current simulation results are in good agreement with the results from the empirical formula. The semi-empirical formula is expressed as follows:

(7) $$\frac{u_{m}}{u_{e}}=A\sqrt{\frac{\rho_{e}}{\rho_{a}}}\cdot \frac{d_{e}}{H}$$

where $u_{m}$ is the jet centerline velocity (m/s), $u_{e}$ is the jet velocity at the nozzle exit (m/s), A is the proportionality constant, $\rho_{e}$ is the jet density at the nozzle exit (kg/m^3^), $\rho_{a}$ is the ambient gas density (kg/m^3^), H is the axial distance from the nozzle exit (m), and $d_{e}$ is the nozzle exit diameter (m).

Furthermore, to address the compressibility effects and the complex shock-wave structures near the nozzle exit, the static pressure distribution along the central jet axis was extracted and is presented in Figure 4b. The pressure profile clearly illustrates the alternating compression and expansion waves (shock diamond structures) characteristic of non-ideal supersonic expansion under the high inlet total pressure. The observed periodic pressure fluctuations (the oscillation between positive and negative gauge pressures) confirm that the turbulence and compressibility models utilized in this study provide sufficient numerical resolution to resolve the aerodynamic characteristics, satisfying the requirements for high-fidelity simulation of supersonic multi-hole jets. 

## 3. Results and Discussion

### 3.1. Analysis of Fluid Dynamic Characteristics of the Jet

#### 3.1.1. Kinetic Energy Attenuation Characteristics of the Jet

**Figure 5:**
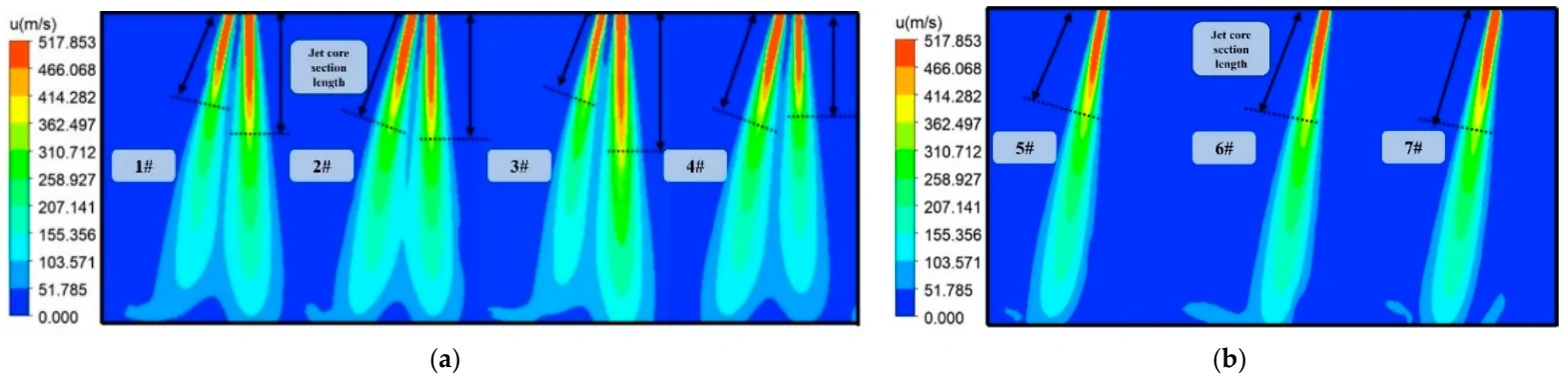
Jet velocity contour map: (**a**) “3+1” hole structures with a central nozzle; (**b**) 3-hole structures without a central nozzle (1#–4# adopt "3+1" hole structures with a central nozzle, and 5#–7# are 3-hole structures without a central nozzle).

The core region (defined as the section from the jet exit to the location where the velocity on the jet centerline decays to the sonic velocity) reflects the kinetic energy preservation capability of the oxygen jet after exiting the nozzle [12,26]; the jet velocity contours for the various nozzles are presented in Figure 5.

**Figure 6:**
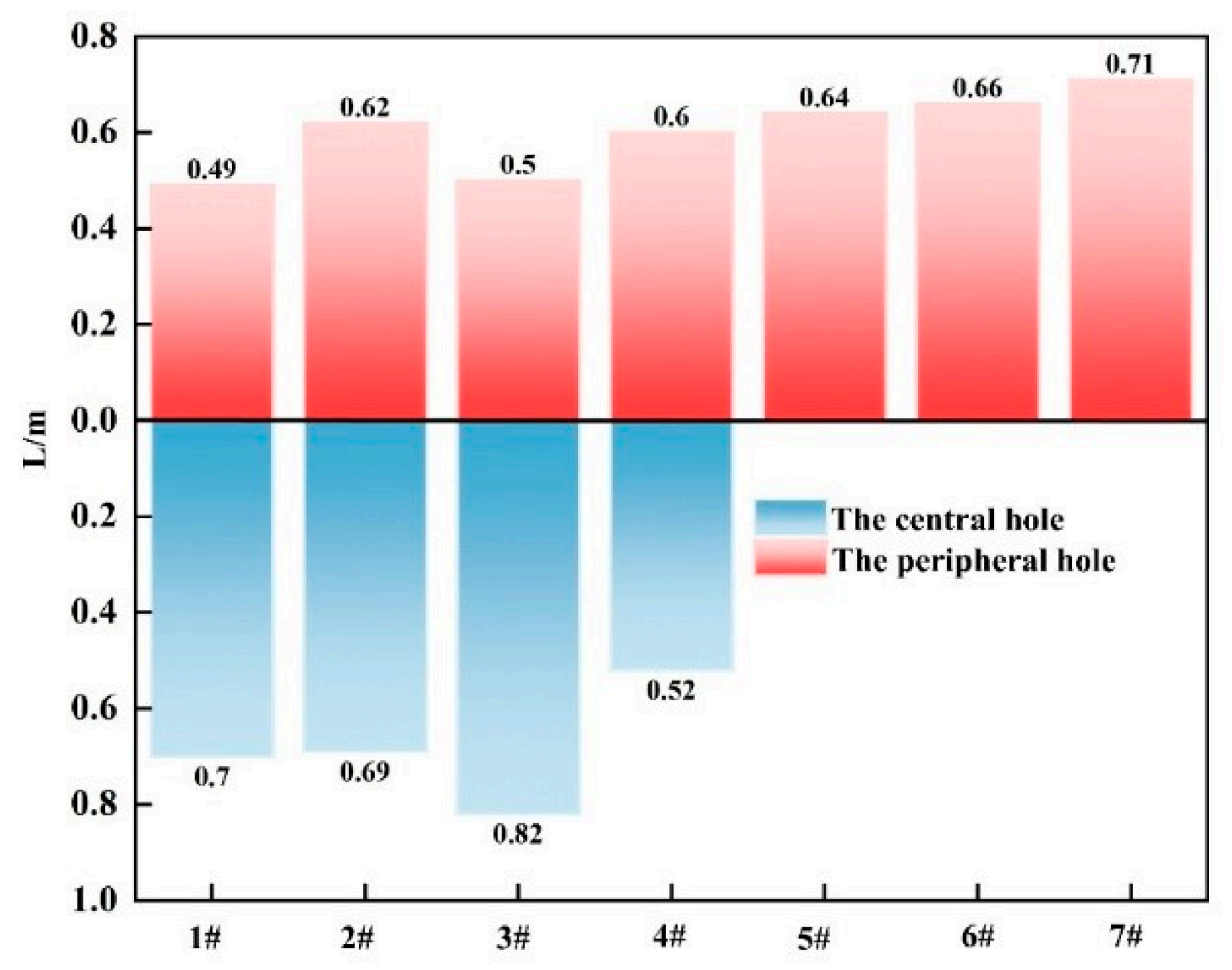
Jet potential core length of different oxygen lance configurations.

The simulation results of the jet potential core length are illustrated in Figure 6, where the positive and negative values represent the core region lengths of the peripheral and central nozzles, respectively. For the 1# oxygen lance, the potential core length of the central nozzle is 0.21 m longer than that of the peripheral nozzles, indicating that the kinetic energy attenuation of the peripheral jets occurs more rapidly than that of the central jet. This is attributed to the fact that, at a total oxygen flow rate of 25,000 ${\;m}^{3}/h$, the oxygen flow rate allocated to the central nozzle of the 1# lance is 2670 ${\;m}^{3}/h$ higher than that of a single peripheral nozzle, thereby enhancing the aerodynamic interference exerted by the central jet on the peripheral jets.

**Figure 7:**
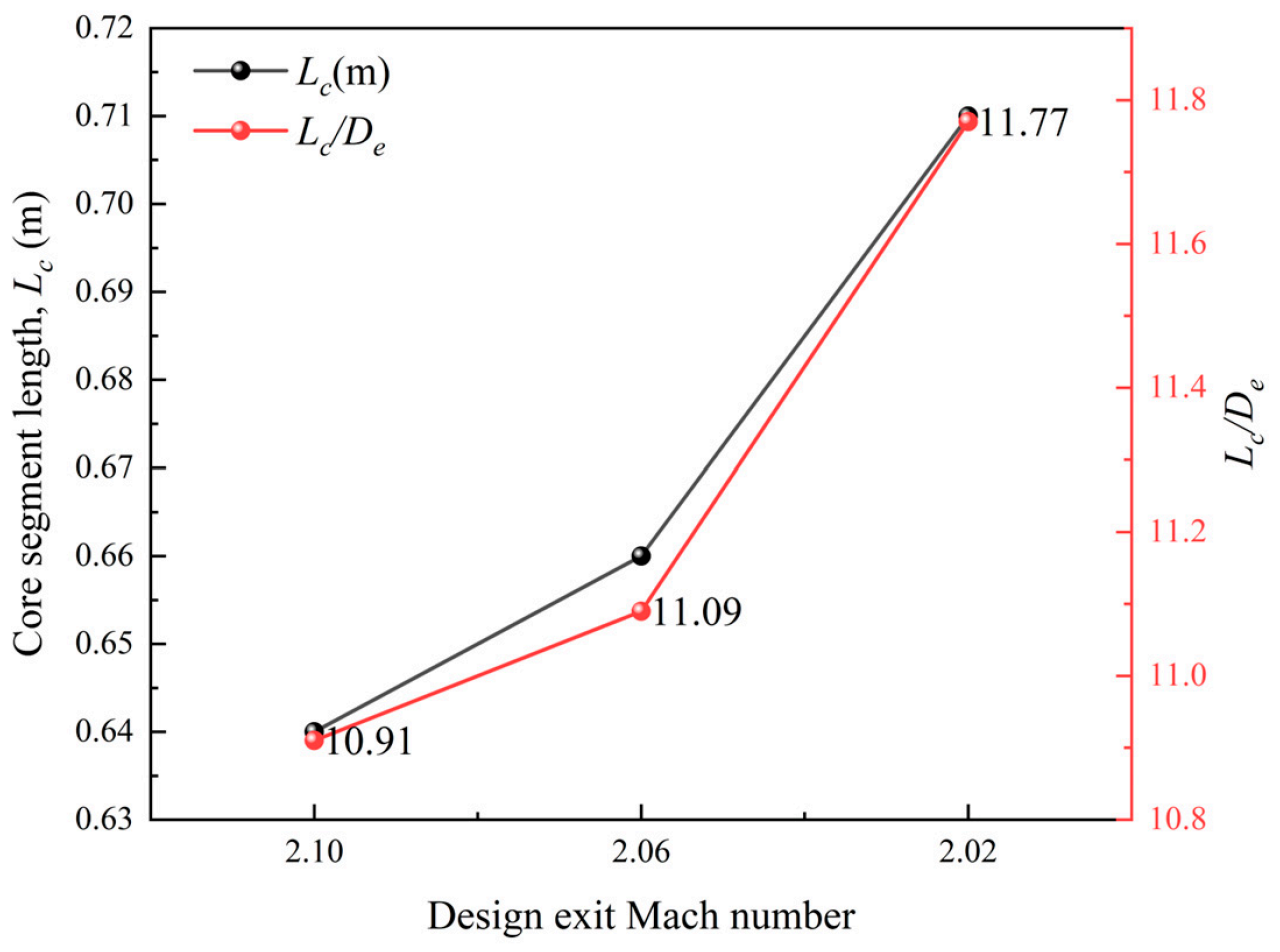
Relationship between design exit Mach number and absolute/dimensionless potential core length.

To investigate the hydrodynamic mechanisms of nozzle structure on jet attenuation, the dimensionless potential core length was introduced to eliminate the physical influence of varying nozzle geometric dimensions. Figure 7 illustrates the variation in the potential core length for traditional three-hole oxygen lances without a central hole (Schemes 5#–7#) under different design exit Mach numbers. As shown, as the design exit Mach number decreases from 2.1 to 2.02, the absolute potential core length increases from 0.64 m to 0.71 m, while the dimensionless potential core length rises from 10.91 to 11.77. From the perspective of supersonic jet theory, under a constant total inlet pressure, reducing the design Mach number shifts the jet from a highly under expanded state toward an ideal expansion state. This transition mitigates the intensity of expansion waves and subsequent oblique shock waves at the nozzle exit. The increase in the dimensionless length indicates that the reduction in aerodynamic energy dissipation within the shock structure decelerates the decay of turbulent kinetic energy, enabling the configuration without a central hole to maintain a high-velocity jet state over a greater distance [26].

**Figure 8:**
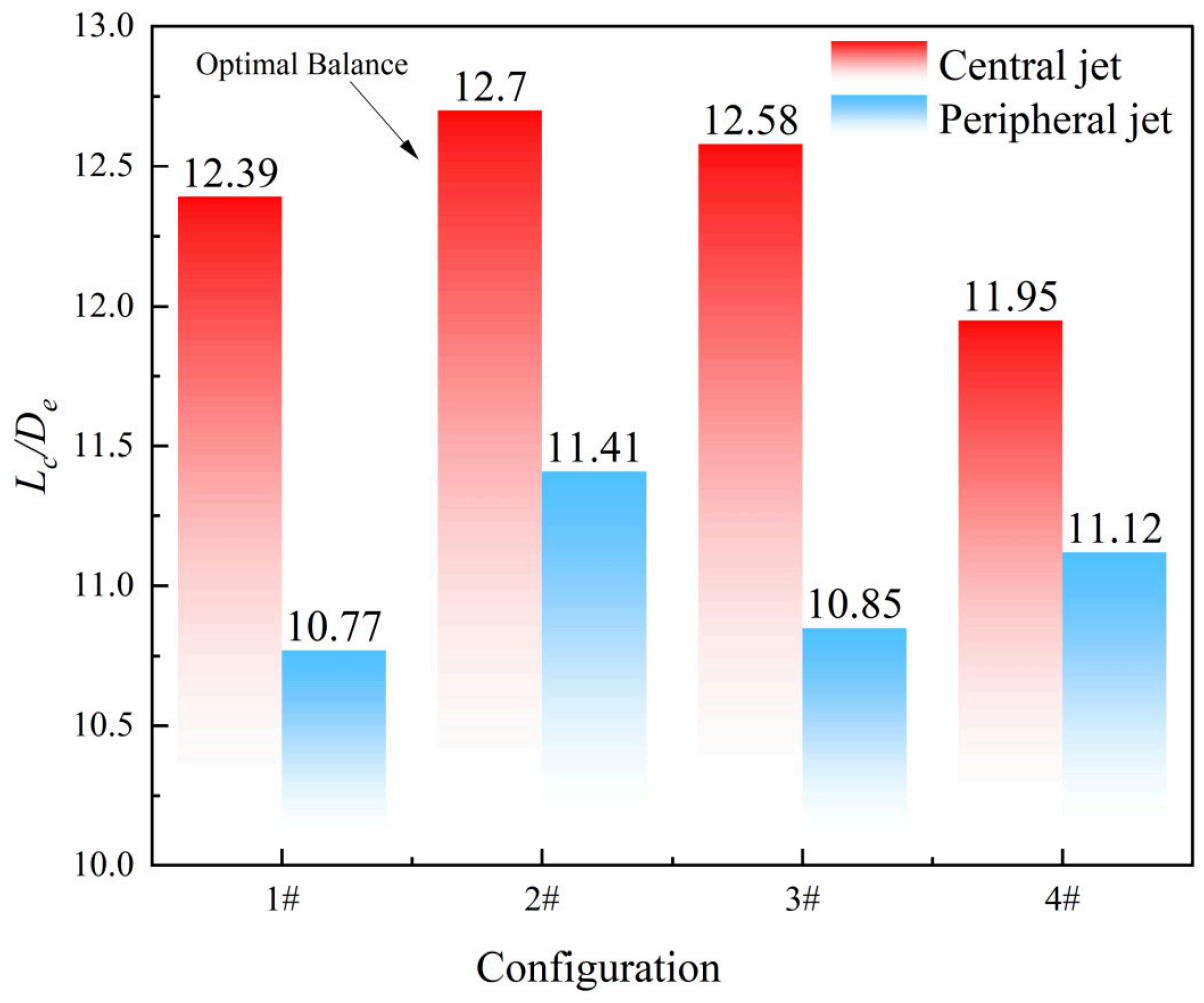
Comparison of dimensionless potential core lengths for oxygen lances with a central hole.

Regarding the “3+1” hole structures with a central nozzle (Schemes 1#–4#), Figure 8 compares the dimensionless potential core lengths of each configuration under a constant total oxygen flow rate and a design Mach number of 2.06. According to the absolute core lengths (Figure 6), the potential core length of the central jet increases with the increment of the oxygen flow rate allocated to it, with Scheme 3# achieving the maximum length of 0.82 m. However, the dimensionless analysis presented in Figure 8 demonstrates that, after eliminating the influence of nozzle exit diameters, Scheme 2# exhibits the highest dimensionless potential core lengths for both the central jet (12.70) and peripheral jets (11.41). This suggests that the balanced 1:1 flow distribution between the central and individual peripheral nozzles in Scheme 2# represents an optimal probing ratio. The objective of this specific distribution is not simply to minimize the mass flow discrepancy between adjacent jets, but rather to establish a precise dynamic balance. Such a flow field distribution effectively ensures that the central jet possesses sufficient momentum to resist severe inward deflection, while the peripheral jets maintain adequate kinetic energy for radial expansion.

**Figure 9:**
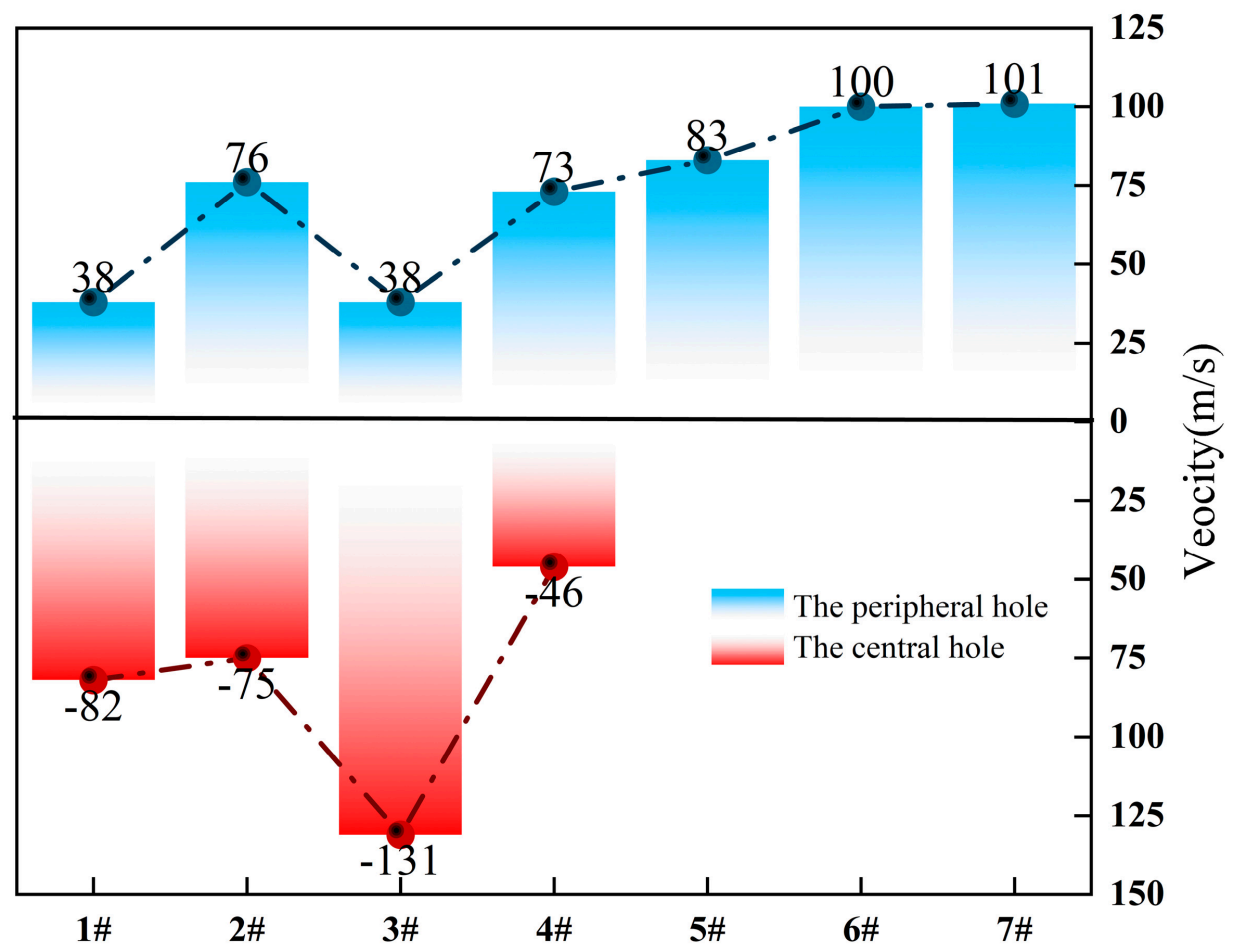
Jet velocity distribution at the molten steel surface for different oxygen lance configurations.

The terminal velocity of the jet upon reaching the liquid surface was further analyzed, as illustrated in Figure 9. During the top-blown converter smelting process, the supersonic jet must possess sufficient dynamic pressure upon reaching the gas–liquid interface to overcome the surface tension of the molten metal and induce droplet generation (splashing) for effective stirring. According to the Kelvin-Helmholtz instability theory for gas–liquid interfaces [26], the critical relative velocity $v_{crit}$ required to trigger surface instability and droplet tearing can be determined by the following theoretical equation:

(8) $$v_{crit}\approx {\left(\frac{4\sigma g\rho_{l}}{\rho_{g}^{2}}\right)}^{1/4}$$

where g is the gravitational acceleration (9.81 m/s^2^, as configured in Table 2); $\rho_{l}$ and σ are the density and surface tension of the actual vanadium-bearing hot metal, approximated as 7000 kg/m^3^ and 1.5 N/m, respectively, based on relevant metallurgical physicochemical studies [27]; and $\rho_{g}$ is the density of the oxygen jet reaching the high-temperature surface. Incorporating the model assumption in Section 2.1.1 that oxygen is treated as a compressible ideal gas, and considering the typical “low operating temperatures” characteristic of the vanadium extraction process (with a reaction zone temperature of approximately T≈ 1623 K [2]), $\rho_{g}$ is calculated as 0.24 kg/m^3^. Substituting these physical parameters into the theoretical equation yields a critical terminal velocity of approximately 51.75 m/s. Therefore, this study adopts 51.75 m/s as the critical velocity threshold to evaluate whether the jet can provide effective interfacial stirring and macroscopic deep-bath circulation.

Based on this critical threshold (51.75 m/s), the 1# prototype oxygen lance was analyzed; the terminal velocity of its central jet is 82 m/s, which exceeds the threshold. In contrast, the terminal velocity of the peripheral jets is only 38 m/s, failing to reach the theoretical lower limit required for effective stirring. These results indicate that the impact of the 1# oxygen lance on the molten steel interface is primarily provided by the central jet, whereas the stirring kinetic energy of the peripheral jets is insufficient.

For the “3+1” configurations with a central nozzle (Schemes 2#–4#), variations in the flow distribution ratio resulted in distinct evolutionary patterns for the terminal velocities. Regarding the central jet, Scheme 4# exhibited the lowest terminal velocity; Scheme 3# achieved the highest terminal velocity of 131 m/s, representing a 59.7% increase compared to Scheme 1#. Regarding the peripheral jets, the terminal velocity of Scheme 3# decreased to 38 m/s, which is identical to that of Scheme 1# and remains below the critical threshold, suggesting that its peripheral jets cannot provide effective collaborative stirring. In contrast, the terminal velocities of the peripheral jets in Schemes 2# and 4# increased by 100% and 92.1% compared to Scheme 1#, respectively, enabling these peripheral jets to provide effective collaborative stirring. This confirms the importance of determining a reasonable flow distribution ratio: it is necessary to consider the effective stirring limits of the peripheral nozzles while ensuring the deep penetration capability of the central nozzle.

**Figure 10:**
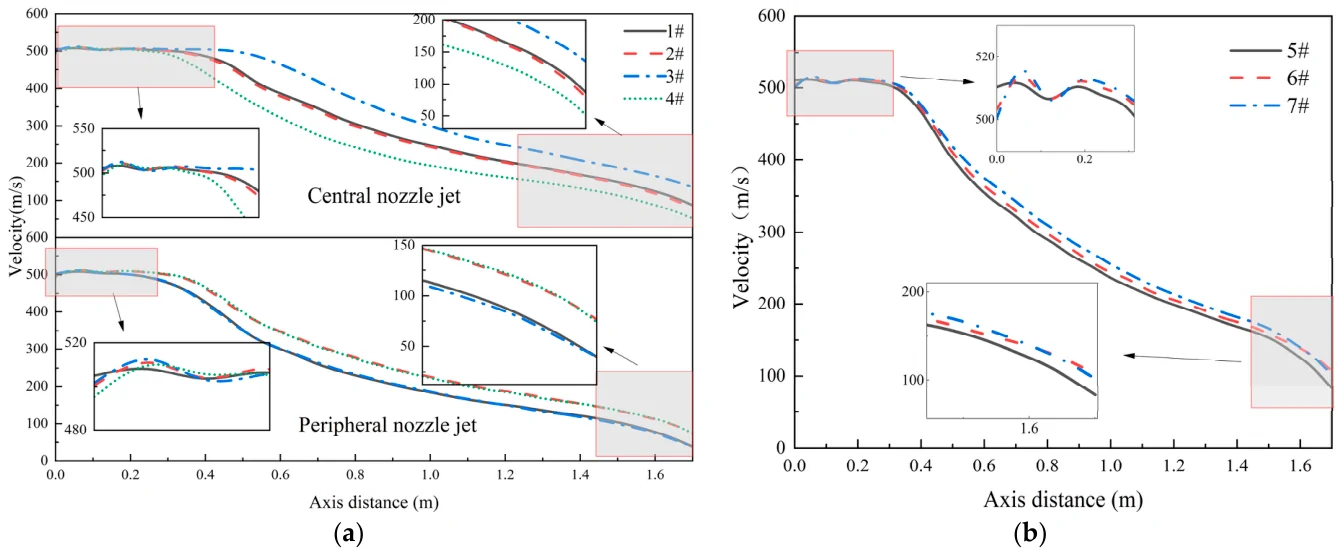
Axial jet velocity distributions of different oxygen lances: (**a**) Jet velocities in the “3+1” hole structures with a central nozzle; (**b**) Jet velocities in the 3-hole structures without a central nozzle.

The axial variation in the jet velocity along the nozzle centerline is illustrated in Figure 10. As observed in Figure 10, both the central and peripheral oxygen jets undergo instantaneous gas expansion within a short distance from the nozzle exit (<0.3 m), resulting in local velocity fluctuations. For the 3-hole structures without a central nozzle (Schemes 5#–7#), the axial velocity distribution patterns corroborate the previous analysis of the dimensionless potential core; specifically, reducing the design Mach number helps mitigate non-ideal expansion effects at the exit and reduces shock-induced energy dissipation, thereby effectively slowing kinetic energy decay. Among the “3+1” hole structures with a central nozzle (Schemes 1#–4#), the decay trends of Schemes 2# and 4#, as well as Schemes 1# and 3#, remain consistent with each other. The ability to maintain identical velocity decay gradients under lower oxygen flow rates indicates that Schemes 2# and 4# possess a superior effective reach in the vertical direction, making them better suited for the high operational lance heights (e.g., 1.9 m) required during the converter vanadium extraction process. 

**Figure 11:**
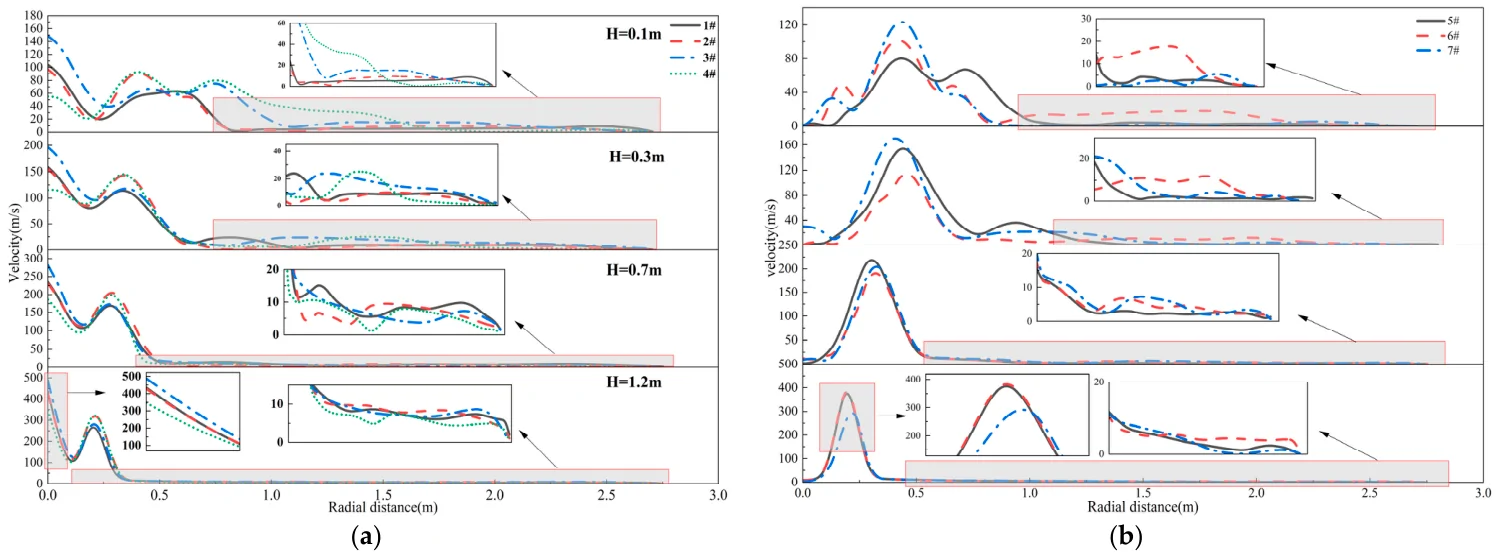
Radial jet velocity distribution of different oxygen lances: (**a**) Jet velocities in the “3+1” hole structures with a central nozzle; (**b**) Jet velocities in the 3-hole structures without a central nozzle.

**Figure 12:**
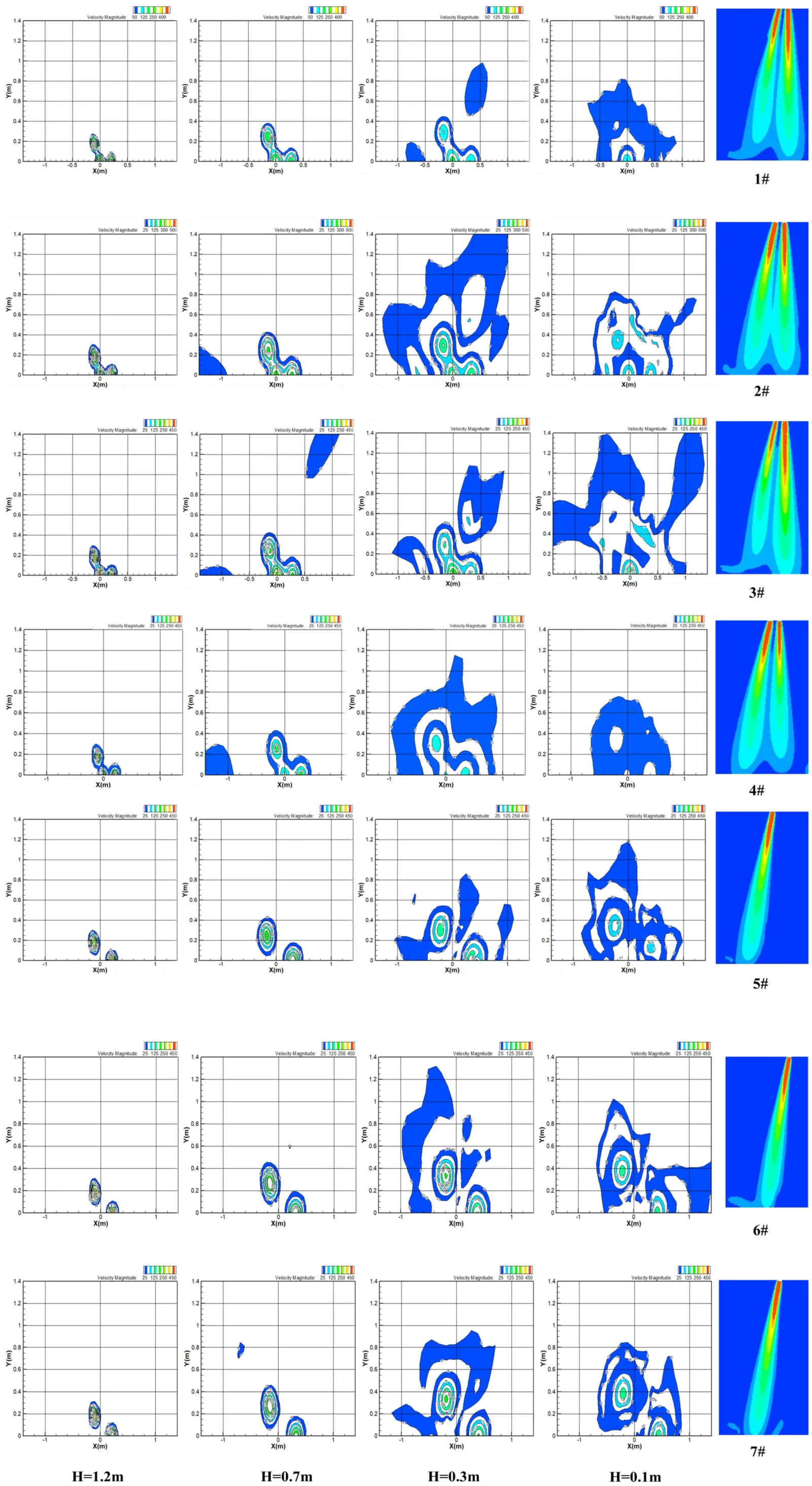
Cross-sectional velocity contours of the jet. (It can be deleted; the icon numbering has been modified).

To characterize the variation in the jet cross-sectional area with axial height, the radial velocity distributions (Figure 11) and velocity contours (Figure 12) of H = 0.1, 0.3, 0.7, and 1.2 m were extracted. Figure 11a illustrates the radial velocity variations for the configurations with a central hole (Schemes 1#–4#). The velocity profiles are characterized by a central peak and surrounding peripheral peaks. As the jet progresses downward (corresponding to a decrease in H), entrainment and momentum exchange between the jet and the ambient gas result in a substantial attenuation of the peak velocities. Concurrently, the peripheral peaks shift outward, signifying the radial expansion of the jet.

A comparison of the radial profiles of Schemes 1#–4# reveals that Scheme 3# consistently exhibits the highest central peak velocity, indicating that it possesses the strongest axial penetrating kinetic energy. Conversely, in the region near the bath surface (H = 0.1 m), the peripheral jet peaks of Scheme 4# deviate the furthest from the center, suggesting that it provides the widest radial coverage area. However, such extreme behaviors are precisely what must be avoided in multi-objective optimization: an excessively strong central jet (Scheme 3#) excessively entrains the surrounding gas, thereby restricting lateral coverage; conversely, an overly broad lateral distribution (Scheme 4#) leads to a deficiency in vertical stirring kinetic energy.

Figure 11b illustrates the flow field characteristics for the configurations without a central hole (Schemes 5#–7#). Distinct from the “3+1” structure, a pronounced low-velocity zone exists at the central axis (r = 0) in these radial velocity profiles. As the axial distance increases, the velocity near the central axis exhibits a relative upward trend, indicating that the multiple independent jets undergo an inward-contracting coalescence phenomenon driven by the pressure difference between the internal and external regions. Comparative analysis reveals that although Scheme 7# possesses the lowest initial kinetic energy at the exit, its low-Mach-number design minimizes turbulent dissipation; consequently, it achieves the highest peak velocity upon reaching the liquid surface, effectively delaying aerodynamic interference between the jets. Conversely, Scheme 6# exhibits outward divergence at the radial extremities, resulting in severe momentum attenuation.

#### 3.1.2. Jet Coalescence Behavior

**Figure 13:**
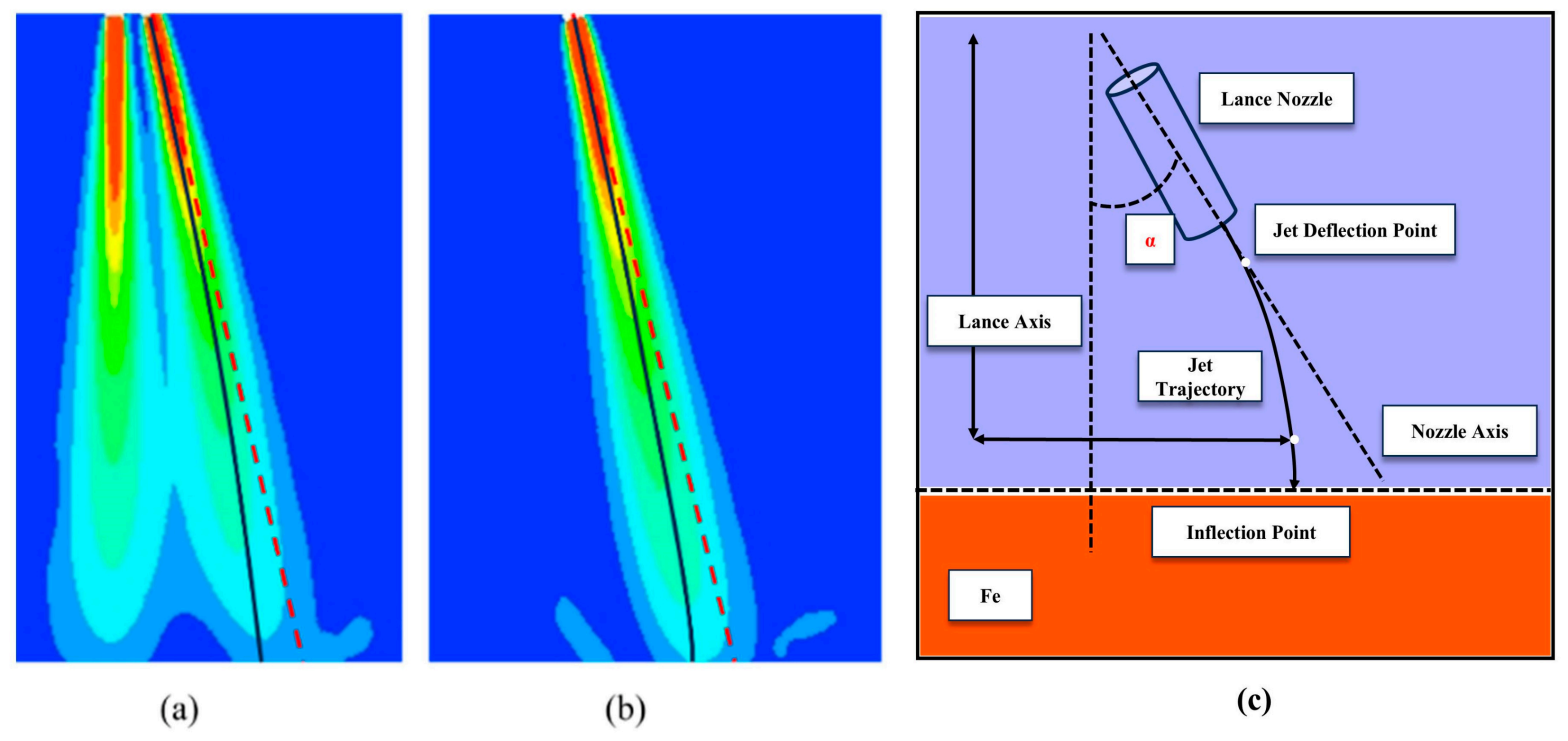
Schematic of jet coalescence behavior and geometric definitions: (**a**) jet velocity contour illustrating the coalescence in the “3+1” hole structure with a central nozzle; (**b**) jet velocity contour illustrating the coalescence in the 3-hole structure without a central nozzle; (**c**) geometric definitions of the jet trajectory and deflection parameters. The background colors in subfigure (**c**) denote the multiphase environment, where the purple region represents the gas phase and the orange region represents the molten steel bath (Fe).

Within a certain distance from the nozzle exit, the supersonic jets essentially travel along their geometric axes (as illustrated in Figure 13). However, as the axial distance of the jet increases, mutual entrainment occurs between the multiple high-speed gas streams, resulting in the formation of a local negative pressure zone in the central region. Driven by the radial pressure gradient, the peripheral jets deviate from their initial axes and converge toward the center; this aerodynamic interference phenomenon is defined as jet coalescence [15]. Since the present numerical model incorporates a molten steel bath, the coalescence process terminates when the jets impact the gas–liquid interface. Consequently, a cross-section at H = 2 m was selected as the uniform reference plane for evaluating the jet offset (ΔX).

**Figure 14:**
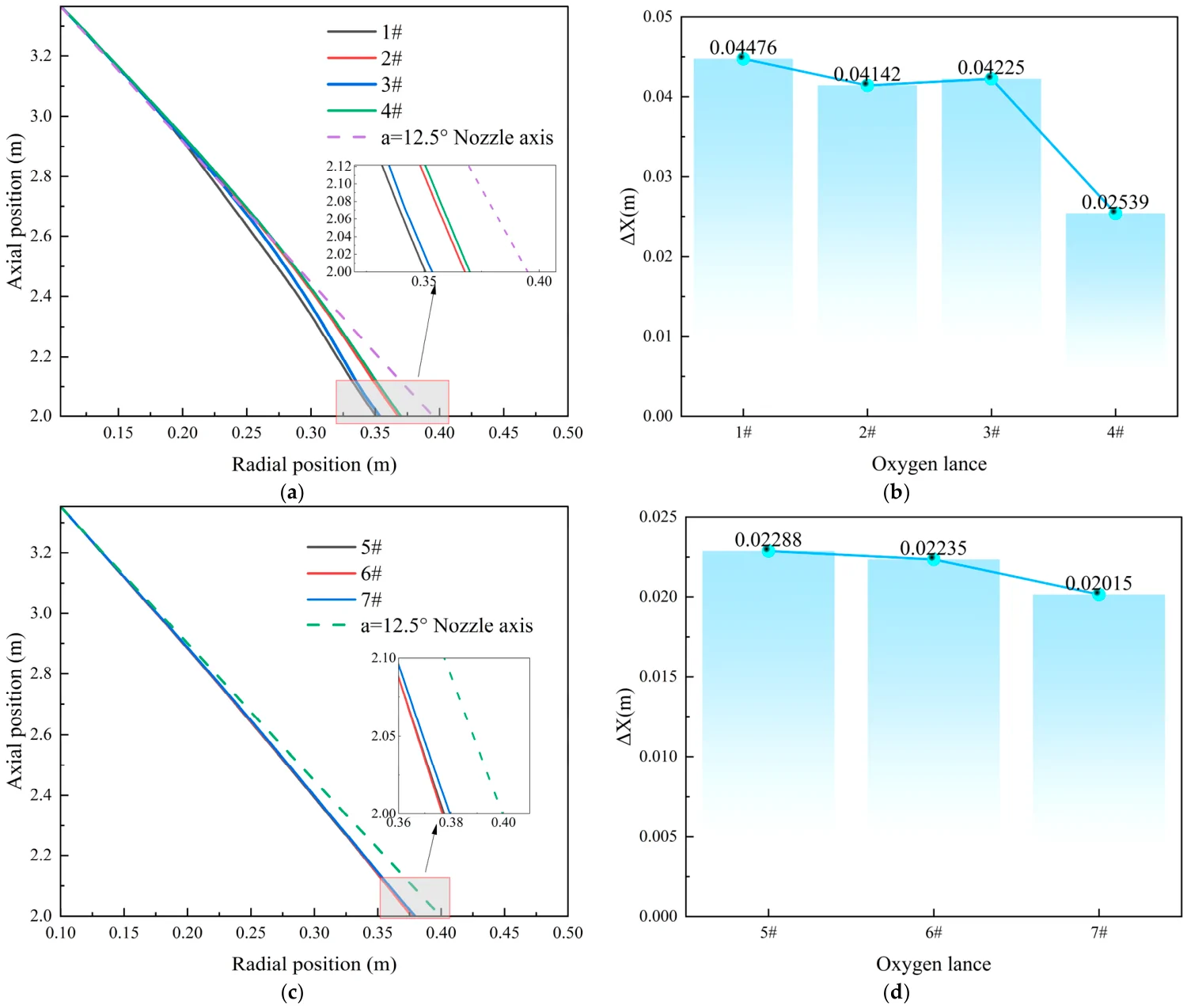
Schematic diagrams of jet deflection for different oxygen lances: (**a**) Jet deflection curves for the 1#–4# lances; (**b**) Jet deflection offsets for the 1#–4# lances; (**c**) Jet deflection curves for the 5#–7# lances; (**d**) Jet deflection offsets for the 5#–7# lances.

The jet center trajectory offsets for the various oxygen lance configurations are presented in Figure 14. For the “3+1” hole structures with a central nozzle utilizing optimized flow distribution (Schemes 2#–4#), the coalescence phenomenon is mitigated compared to that of the 1# prototype. Scheme 4# exhibits the minimum offset (0.02539 m), representing a 43.2% reduction compared to Scheme 1#. This indicates that under a constant design Mach number, the flow rate of the central hole is the primary factor influencing coalescence. In Scheme 4#, the reduction in the central flow rate decreases the inward radial pressure gradient, thereby resulting in the minimum offset. Based on the previous analysis of kinetic energy attenuation, this effect is achieved at the expense of decreasing the effective penetration velocity of the central jet. Scheme 2# adopts an equal flow distribution ratio; while ensuring the penetration capability of the central jet, it controls the offset to 0.04142 m (a 7.5% reduction compared to the prototype), which can simultaneously satisfy the requirements of suppressing jet deflection and enhancing deep bath penetration.

For conventional 3-hole structures without a central nozzle (Schemes 5#–7#), the jet offsets (approximately 0.020–0.022 m) are overall lower than those of the configurations with a central hole. This indicates that the absence of a central hole structure and the resulting increase in single-nozzle flow rates enhance the axial momentum of the jets, effectively weakening the radial converging forces. When the design exit Mach number is reduced from 2.10 (Scheme 5#) to 2.02 (Scheme 7#), the jet offset decreases from 0.02288 m to 0.02015 m. This is attributed to the fact that the low-Mach-number condition mitigates the energy dissipation caused by shock waves at the nozzle exit, allowing the overall jet morphology to maintain better stability.

### 3.2. Evolutionary Patterns and Morphological Characteristics of the Impact Cavity

**Figure 15:**
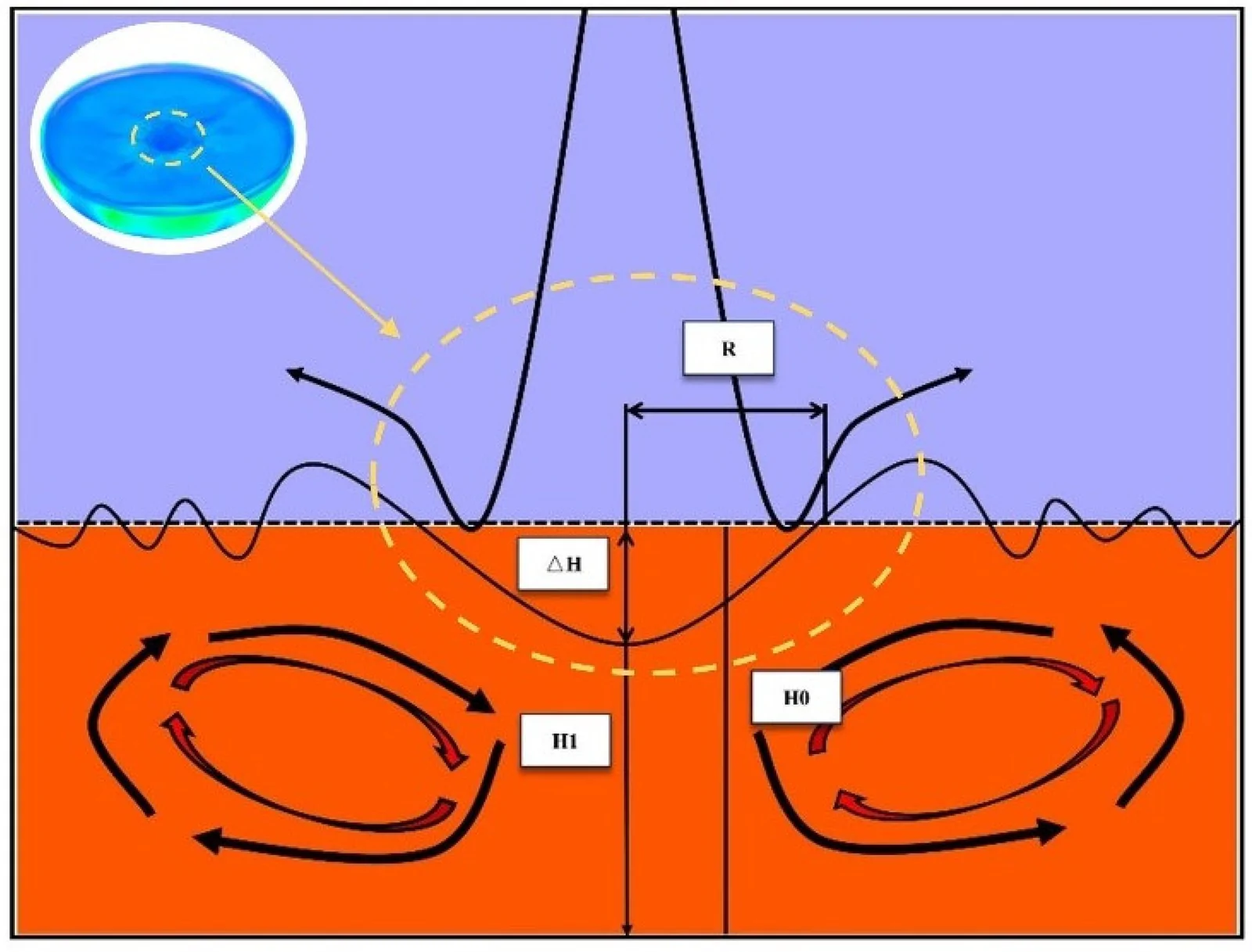
Schematic diagram of the impact cavity morphology.

When the supersonic oxygen jet impacts the molten steel bath, the dynamic pressure of the gas stream displaces the liquid at the gas–liquid interface, forming an impact cavity, as shown in Figure 15. The impact depth (h) and impact radius (r) data extracted from the simulation interval of 1.0–2.0 s (Table 5 and Table 6) reveal that both characteristic parameters exhibit dynamic periodic oscillations. As reported in reference [28], the perturbation of the gas–liquid interface is closely associated with the periodic oscillation of the impact cavity; the dynamic evolution of its morphology is essentially governed by the alternating variations in the macroscopic flow field within the molten bath.

**Figure 16:**
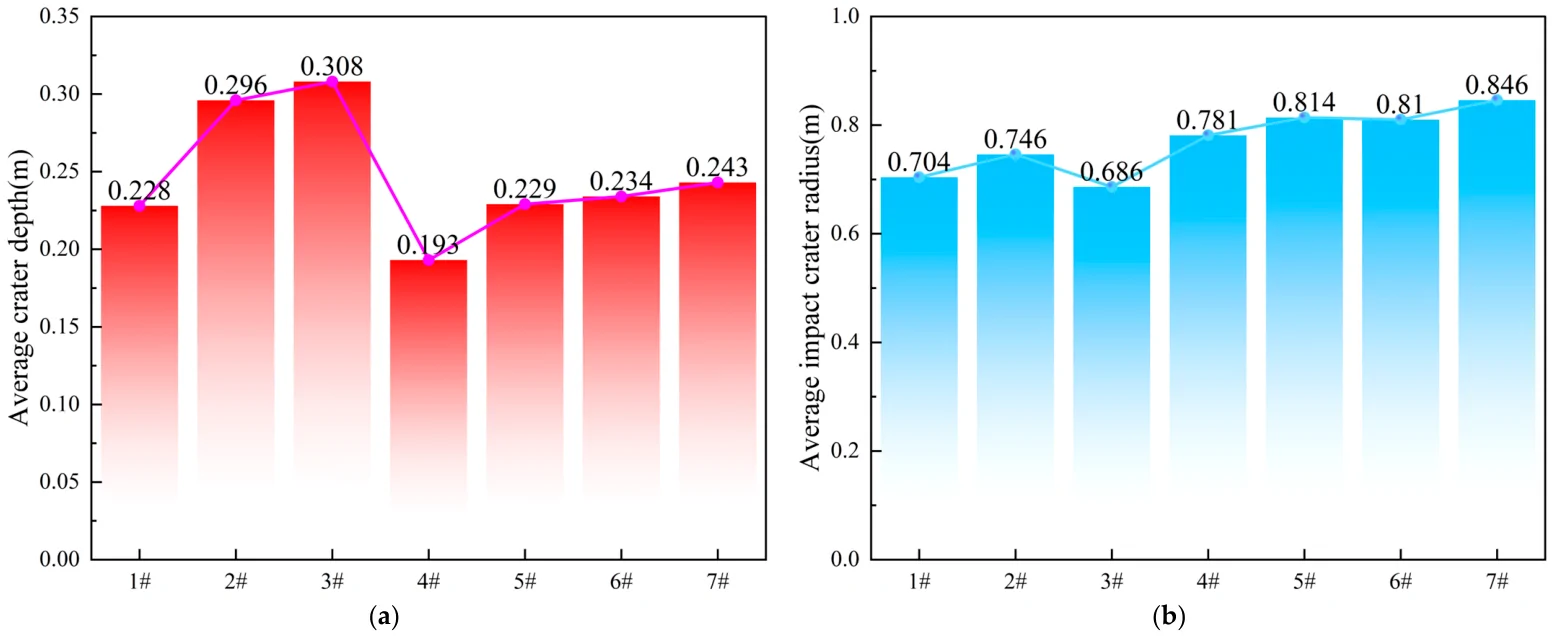
Morphological characteristic parameters of the impact cavity: (**a**) average impact depth; (**b**) average impact radius.

The average impact depth and radius within this time interval were statistically analyzed (Figure 16). Among the optimized configurations with a central hole, specifically Schemes 2#, 3#, and 4#, an inverse constraint relationship is observed between the impact depth and the impact radius. Scheme 3# exhibits the maximum impact depth (0.308 m) but the minimum impact radius (0.686 m); conversely, Scheme 4# achieves the largest impact radius (0.781 m), while its impact depth decreases to the minimum value (0.193 m). This behavior is governed by the momentum distribution between the central and peripheral jets, which is a critical consideration in the optimization of the vanadium extraction process. When an excessive portion of total momentum is allocated to the central hole (as in Scheme 3#), the central jet induces an inward entrainment effect on the surrounding jets, thereby restricting lateral expansion [16]. Conversely (as in Scheme 4#), when the kinetic energy of the peripheral jets dominates, radial expansion is enhanced at the expense of weakened central impact energy. Furthermore, for the conventional 3-hole structures without a central nozzle, denoted as Schemes 5#–7#, the impact depths range from 0.22 to 0.24 m, which is essentially comparable to the 1# prototype. Due to the absence of the inward entrainment effect from a central jet, their impact radii are larger than those of the configurations with a central hole.

To evaluate the gas–liquid reaction interface, the morphology of the impact cavity is typically simplified as a paraboloid of revolution. Based on the extracted impact radius (r) and impact depth (h), the formula for the surface area (S) of the cavity is as follows:

(9) $$S=\frac{\pi r}{6h^{2}}\left[{\left(r^{2}+4h^{2}\right)}^{\frac{3}{2}}-r^{3}\right]$$

where S is the surface area of the cavity formed by jet impingement (the gas–liquid reaction interface area), $m^{2}$; r is the surface radius of the impact cavity, m; and h is the maximum impact depth of the cavity formed by the jet penetrating the liquid surface, m. It should be noted that the actual simulated gas–liquid interface exhibits intense transient deformation, localized splashing, and strong spatial asymmetry under supersonic impingement. The idealized paraboloid approximation (Equation (9)), which relies on the time-averaged impact depth and radius, is not intended to precisely track the highly wrinkled instantaneous phase boundary. Instead, it is utilized here strictly as a macroscopic, time-averaged geometric baseline. This simplification provides a mathematically stable and normalized index to quantitatively compare the relative expansion of the effective gas–liquid reaction area among the different lance configurations.

**Figure 17:**
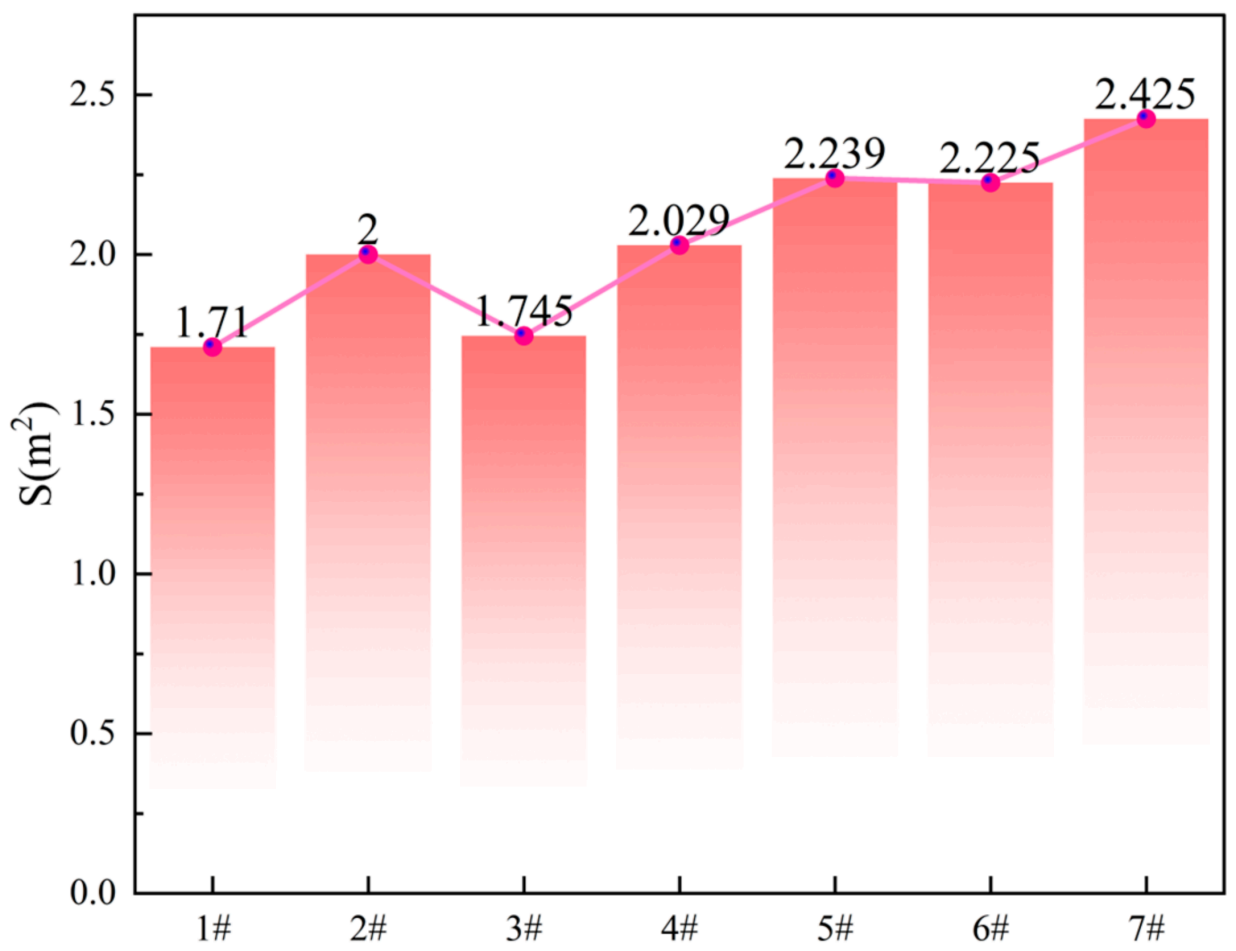
Comparison of average impact cavity surface areas for different oxygen lances.

The statistical results of the calculated average surface area are illustrated in Figure 17. Among the “3+1” hole structures with a central nozzle (Schemes 1#–4#), the ranking of the surface area (4# > 2# > 3# > 1#) is consistent with that of the impact radius. Based on geometric relationships, it can be inferred that for an impact cavity approximating a paraboloid of revolution, the radial distance exerts a more significant influence on the increase in surface area. For instance, although Scheme 3# possesses the deepest penetration capability, the entrainment effect results in a “deep and narrow” cavity morphology with a surface area of only 1.745 $m^{2}$; conversely, while Scheme 4# achieves the largest surface area in this group (2.029 $m^{2}$), its depth is insufficient for deep-bath stirring. Among the 3-hole structures without a central nozzle (Schemes 5#–7#), Scheme 7# achieves the absolute maximum surface area (2.425 m^2^) across all design schemes due to the absence of the central entrainment effect.

Based on the evolution of the morphological characteristic parameters described above, the smelting process in the vanadium extraction converter imposes multi-objective requirements on the impact cavity morphology. It necessitates both an extensive surface area (e.g., Schemes 7# or 4#) to provide a sufficient gas–liquid reaction interface for promoting decarburization and vanadium extraction, and an adequate impact depth (e.g., Scheme 3#) to ensure effective deep-bath stirring. However, the maximization of a parameter in one dimension often leads to a reduction in another. Therefore, the core objective of this study is not to maximize the values of individual schemes, but rather to identify the optimal aerodynamic balance.

### 3.3. Analysis of Internal Flow Characteristics Within the Molten Bath

#### 3.3.1. Spatial Distribution Characteristics of the Internal Velocity Field

**Figure 18:**
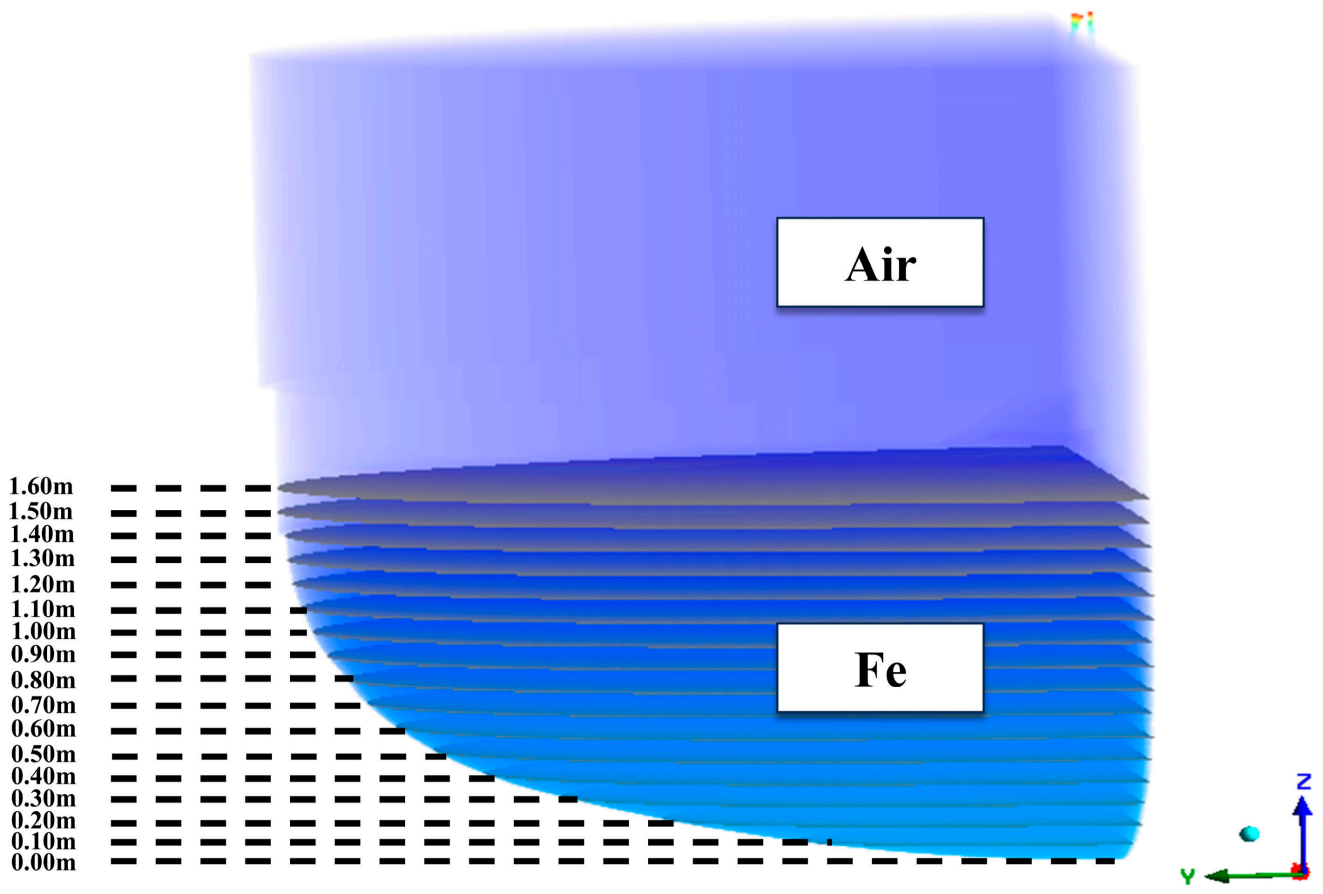
Locations of the horizontal cross-sections.

To evaluate the influence of various oxygen lance nozzle configurations on the macroscopic flow characteristics of the molten bath, 17 characteristic horizontal cross-sections were uniformly extracted along the bath depth, as illustrated in Figure 18.

**Figure 19:**
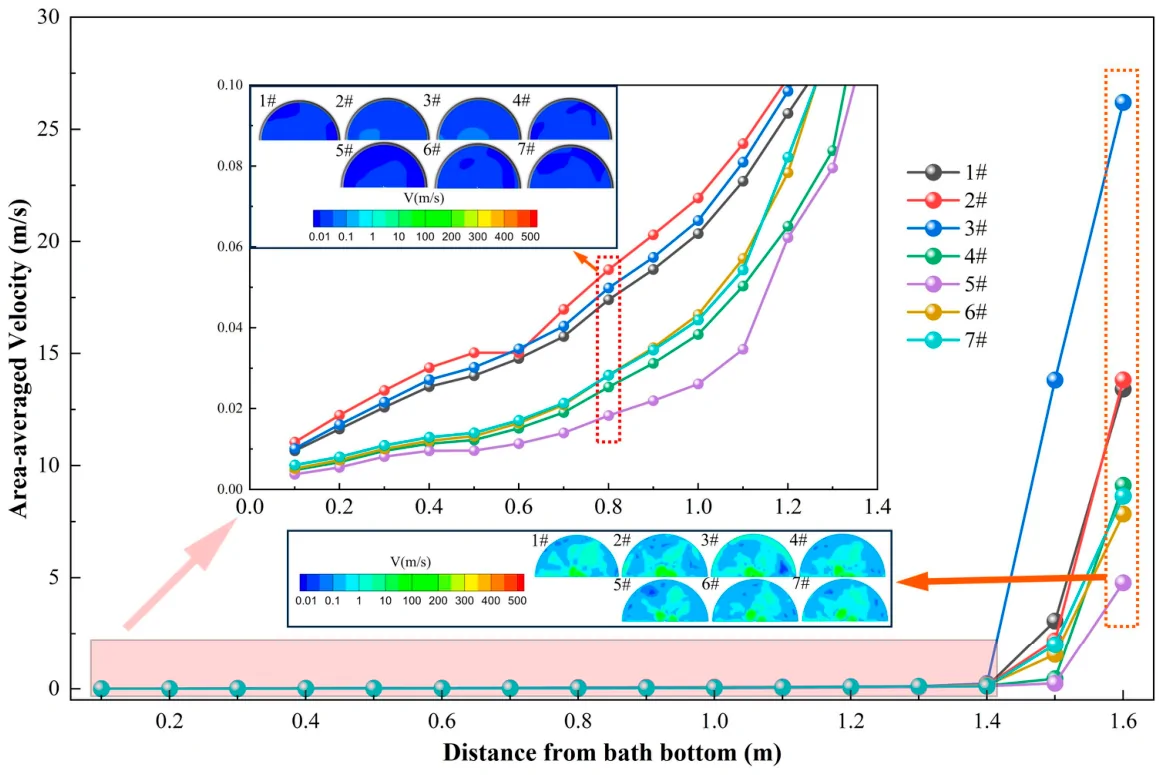
Area-weighted average velocity of horizontal cross-sections at different distances from the converter bottom.

Under all oxygen lance configurations, the area-weighted average flow velocity of the horizontal cross-sections in the molten bath exhibits a characteristic gradient decay along the depth direction (Figure 19). At the gas–liquid interface (1.6 m), the fluid attains maximum initial kinetic energy due to the dynamic pressure impact of the supersonic jets, resulting in a peak velocity. As the depth increases, the jet kinetic energy is gradually dissipated through momentum exchange between the gas–liquid phases and within the liquid layers, until the average velocity drops to an extremely low level (approximately 0.01 m/s) at the bottom of the bath (0.1 m). A comparison between the two main categories of nozzle structures reveals that the “3+1” hole structures with a central nozzle (Schemes 1#–4#) provide overall superior hydrodynamic conditions compared to the 3-hole structures without a central nozzle (Schemes 5#–7#). Taking the near-bottom cross-section at 0.1 m as an example, the average velocities for Schemes 1#, 2#, and 3# are 0.00958 m/s, 0.01175 m/s, and 0.01014 m/s, respectively—values consistently higher than the 0.00379–0.00605 m/s observed for Schemes 5#–7#. These results indicate that the introduction of a central jet provides longitudinal penetrating kinetic energy, thereby reducing the low-velocity dead zones at the bottom [18].

Further analysis of the internal flow field distribution for the “3+1” hole structures with a central nozzle shows that Scheme 3# exhibits a significantly higher average velocity at the 1.6 m surface (26.20 m/s) compared to Scheme 1# (13.42 m/s) and Scheme 2# (13.83 m/s). Correlating this with the previous morphological analysis, this is attributed to the excessive oxygen flow rate allocated to the central hole in Scheme 3#, which causes the jet kinetic energy to be concentrated primarily at the bath surface. This local energy concentration fails to be effectively converted into deep penetration force; instead, it intensifies the dissipation of kinetic energy at the surface, potentially increasing the risk of molten steel splashing. In contrast, while the peak surface velocity of Scheme 2# is not the highest, it maintains the most optimized and stable velocity distribution throughout the lower depth interval (0.1 m to 1.4 m) within the group. This suggests that the balanced 1:1 flow distribution effectively reduces kinetic energy dissipation at the interface, promoting the conversion of jet energy into the longitudinal shear force that drives circulation throughout the entire molten bath.

#### 3.3.2. Evaluation of Stagnant Regions Within the Molten Bath

**Figure 20:**
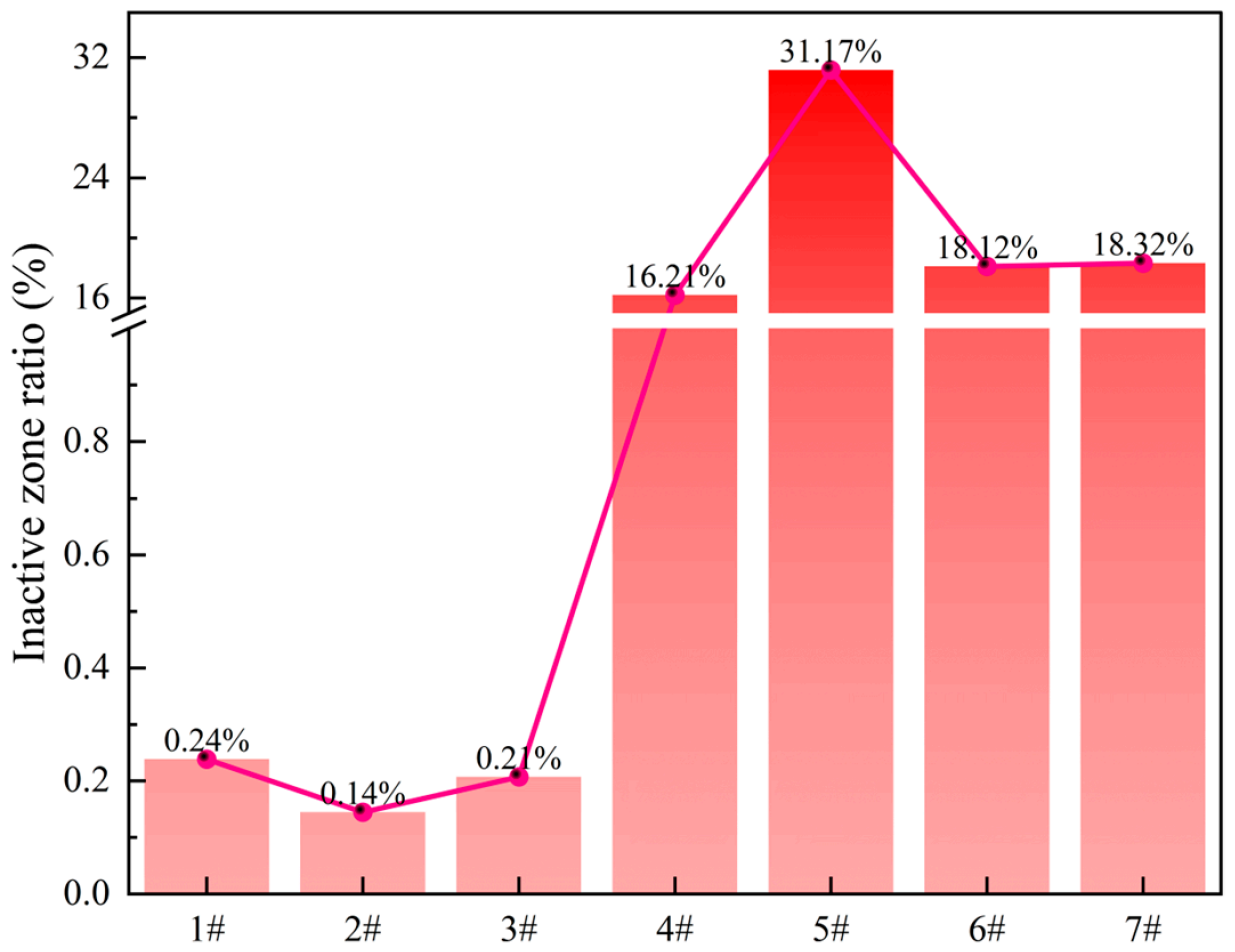
Volume fraction of the “inactive zone” within the top-blown converter for different schemes.

To evaluate the impact of various oxygen lance configurations on the stirring dead zones within the molten bath, the volume fraction of regions with a flow velocity below 0.01 m/s (defined as the inactive zone ratio) was quantified, as illustrated in Figure 20.

The calculation results demonstrate that “3+1” hole structures with a central nozzle possess a distinct advantage in deep-stirring capability, with inactive zone volume fractions consistently maintained below 0.25%. Specifically, Scheme 2#, which adopts a 1:1 flow distribution ratio between the central hole and an individual peripheral nozzle, exhibits the optimal performance. Its inactive zone ratio is minimized to only 0.14%, representing a strict 41.6% relative decrease compared to the 0.24% observed in the 1# prototype. This indicates that a balanced flow distribution maximizes the suppression of collision-induced energy dissipation between the jets; consequently, the kinetic energy from both the central and peripheral jets is effectively transmitted to the deep regions of the molten bath, significantly reducing the volume of the bottom stagnant zone [3,18]. In contrast, when the central hole flow distribution is insufficient (as seen in Scheme 4# with an inactive zone ratio of 16.21%) or when the central hole is entirely omitted (Schemes 5#–7#), the jets struggle to drive the middle and lower fluid layers to form effective macroscopic recirculation due to the deficiency in longitudinal penetrating momentum. Under conditions without bottom blowing, extensive stagnant regions form at the bottom of the bath [29], with inactive zone ratios generally exceeding 18%, reaching as high as 31.17% for Scheme 5#. These findings confirm that the central jet plays a primary role in intensifying the stirring within the deep-bath regions.

### 3.4. Numerical Uncertainty and Sensitivity Analysis

To rigorously quantify the numerical uncertainty and evaluate the robustness of the computational model against operational boundary fluctuations, a systematic sensitivity analysis was conducted. Taking the optimal “3+1” configuration (Case 2#) as the benchmark model, the operating inlet total pressure was deliberately perturbed by ±5% (807,500 Pa and 892,500 Pa, respectively). The transient responses of the critical macroscopic hydrodynamic parameters—specifically, the time-averaged impact depth and the volume fraction of the bottom inactive zone—were continuously quantified.

As detailed in Table 7, under the +5% pressure perturbation, the average impact depth exhibited a minor relative change of 7.8%. Concurrently, the bottom inactive zone ratio shifted with an absolute fluctuation of less than 6.2%. Under the −5% pressure decompression, the absolute deviations for both metrics remained strictly within an acceptable margin. These constrained micro-level variations mathematically demonstrate that the coupled VOF-turbulence numerical framework is highly robust and insensitive to reasonable boundary variations, ensuring excellent reproducibility of the reported fluid dynamic trends.

## 4. Comprehensive Fluid Dynamic Evaluation and Optimal Oxygen Lance Selection

### 4.1. Comprehensive Fluid Dynamic Evaluation of the 3-Hole Structures Without a Central Nozzle

For the conventional 3-hole structures without a central nozzle (Cases 5#, 6#, and 7#), decreasing the design exit Mach number facilitates the transition of the jet towards an ideally expanded state, thereby mitigating the energy dissipation induced by shock waves.

Regarding jet characteristics, a lower Mach number slows down the attenuation of kinetic energy and weakens the inward coalescence and deviation among multiple jets. In terms of the impingement cavity morphology, the gas–liquid reaction surface area increases as the Mach number decreases. Concerning the internal flow field of the molten pool, lowering the Mach number effectively reduces the jet energy loss, leading to a decrease in the volume fraction of the inactive zone at the bottom (e.g., when the Mach number decreases from 2.10 to 2.02, the inactive zone fraction drops from 31.17% in Case 5# to approximately 18% in Case 7#).

Consequently, Case 7# performs best in minimizing shock wave energy dissipation and maximizing the gas–liquid reaction surface area (2.425 m^2^), making it the optimal scheme among the configurations without a central nozzle.

### 4.2. Comprehensive Fluid Dynamic Evaluation of the “3+1” Hole Structures with a Central Nozzle

The core design principle of the oxygen lance with a central nozzle lies in regulating the flow distribution between the central and peripheral nozzles, which subsequently affects the aerodynamic interference among adjacent jets.

In terms of jet characteristics, the flow distribution alters the intensity of aerodynamic interference between adjacent jets [15]. An increase in the flow proportion of the central nozzle enhances the aerodynamic disturbance and entrainment of the peripheral jets, accelerating the overall kinetic energy attenuation. Conversely, while reducing the central nozzle flow can lower the inward radial pressure difference to suppress jet coalescence, it results in insufficient axial penetration kinetic energy for the central jet. Regarding the impingement cavity morphology, flow distribution dictates the inverse restriction relationship between the impingement depth and radius. Excessive momentum of the central jet triggers a more pronounced inward entrainment effect, restricting the lateral expansion of the gas flow and resulting in a “deep and narrow” cavity. Conversely, if the lateral jet momentum dominates, the gas–liquid reaction area can be expanded, but the longitudinal depth will be significantly attenuated, presenting a “shallow and wide” characteristic. Concerning the internal flow field of the molten pool, the flow distribution determines the velocity distribution characteristics along the depth direction and the evolution laws of the inactive zones. Excessive flow distribution to the central nozzle causes the jet kinetic energy to overly concentrate at the liquid surface layer (characterized by high peak surface velocity) and exacerbates dissipation; this energy fails to convert into deep penetration force and increases the risk of splashing. Insufficient central flow distribution, due to the lack of longitudinal penetration momentum, makes it difficult to drive the middle and lower fluid layers to form an effective macroscopic circulation, leading to a large-area stagnation dead zone at the bottom.

Therefore, Case 2#, which adopts a 1:1 balanced flow distribution between the central and a single peripheral nozzle, effectively avoids the adverse effects of imbalanced momentum distribution. This scheme maximizes the suppression of collision dissipation at the interface, maintains an optimal and stable velocity distribution within the deep molten pool region, and controls the bottom dead zone proportion to the lowest level (0.14%, a 41.6% reduction from the prototype), establishing it as the optimal scheme among the “3+1” hole structures with a central nozzle.

### 4.3. Optimal Oxygen Lance Selection

Comparing the best schemes from both groups (Cases 2# and 7#) with the original Case 1#:

Compared to the original Case 1#, Case 2# achieved superior kinetic energy retention in terms of jet characteristics, with the jet coalescence deviation reduced by 7.5%. Regarding the impingement cavity morphology, the gas–liquid reaction surface area increased by 17.0% (reaching 2.000 m^2^), and the impingement depth improved by 29.8%. For the internal flow field of the molten pool, the synergistic agitation of the central and peripheral flows reduced the bottom inactive zone proportion to 0.14% (achieving a 41.6% reduction), indicating that its macroscopic fluid dynamic conditions are overall superior to Case 1#.

Compared to Case 1#, Case 7# performed well in maintaining jet kinetic energy and mitigating coalescence, possessing the globally maximum gas–liquid reaction surface area (approximately 41.8% higher than Case 1#). However, constrained by the absence of a central nozzle, its jet kinetic energy cannot provide effective deep impingement agitation in the central region. In the internal molten pool flow field, its deep agitation capability is inferior to Case 1# and other configurations with a central nozzle, resulting in a bottom inactive zone proportion exceeding 18.0%, thus failing to achieve the goal of comprehensive optimization.

Synthesizing all fluid dynamic indicators, Case 2# (the configuration with a central nozzle and a 1:1 flow distribution ratio between the central and a single peripheral nozzle) is finally selected as the optimal oxygen lance nozzle scheme for the vanadium extraction converter process.

### 4.4. Model Limitations and Future Work

While the current CFD model provides critical fluid dynamic insights, inherent physical limitations exist due to the absence of direct experimental or industrial validation. The extreme operational environment of the 200 t vanadium extraction converter—characterized by opaque gas-slag-metal multiphase flows and high temperatures (approximately 1623 K)—makes in situ measurements of jet deflection, impact cavity morphology, and bottom-zone velocity fields practically unfeasible using current sensor technologies. To mitigate these limitations, our numerical approach was rigorously validated against isentropic theory and Cheslak’s semi-empirical formula to ensure the accurate capture of fundamental aerodynamic energy decay in the supersonic core region. The optimized fluid dynamic laws and structural parameters identified in this study, particularly the 1:1 balanced flow distribution ratio for the “3+1” configuration (Case 2#), establish a robust theoretical framework. In our forthcoming research phase, these numerical findings will be cross-validated through specifically designed scaled physical water modeling and upcoming industrial trials. The macroscopic metallurgical indicators, such as the actual vanadium oxidation rate and the reduction in splashing severity, will be utilized to definitively verify the hydrodynamic optimizations proposed herein.

## 5. Conclusions

In this study, a three-dimensional CFD method was employed to conduct a fluid dynamic optimization study on the multiphase flow process within a 200 t vanadium extraction converter. Under vanadium extraction conditions, by investigating the effects of the nozzle Mach number design and the inter-nozzle flow distribution on the gas–liquid two-phase flow field, the following conclusions are drawn:

(1) Reducing the design Mach number can effectively improve the fluid dynamic performance of conventional 3-hole structures without a central nozzle. As the exit Mach number decreases, the jet shock wave energy dissipation is mitigated, and the kinetic energy attenuation is slowed down. However, restricted by the structure lacking a central nozzle, the proportion of its bottom dead zone remains critically high.
(2) An excessively large flow proportion in the central nozzle triggers strong entrainment, causing the impingement cavity to exhibit a “deep and narrow” characteristic; furthermore, the jet kinetic energy is prone to concentrated dissipation at the surface layer, making it difficult to translate into deep penetration force. Conversely, a deficiency in the central nozzle flow leads to a “shallow and wide” impingement cavity; due to insufficient longitudinal penetration force, it is difficult to drive the deep circulation in the molten pool, resulting in a large-area dead zone at the bottom.
(3) Based on multi-objective optimization, Case 2#, which adopts a 1:1 flow ratio between the central nozzle and a single peripheral nozzle, serves as the optimal oxygen lance nozzle configuration for the vanadium extraction converter.

## Figures and Tables

**Table 1:** Optimized Design Schemes of the Oxygen Lance.

	Throat Diameter (mm)	Outlet Diameter (mm)	Dip Angle (Deg)	Oxygen Flow Rate (m^3^/h)	
1#	41.69/34.29	56.5/45.5	12.5	8252/16,748	2.1/2.05
2#	40.78/40.78	54.33/54.33	12.5	6250/18,750	2.06
3#	48.93/34.6	65.2/46.1	12.5	10,000/15,000	2.06
4#	32.65/40.49	43.5/53.95	12.5	4452.05/20,547.95	2.06
5#	43.29	58.68	12.5	25,000	2.1
6#	44.67	59.51	12.5	25,000	2.06
7#	46.06	60.34	12.5	25,000	2.02

**Table 2:** Operating and boundary conditions.

Parameter	Value	Parameter	Value
1. Operating & Global Settings			
Mass of hot metal	200 t	Total oxygen flow rate	25,000 Nm^3^/h
Lance height	1700 mm	Oxygen supply intensity	1.81 Nm^3^/(t·min)
Operating absolute pressure	101,325 Pa	Inlet oxygen temperature	300 K
Gravitational acceleration	9.81 m/s^2^		
2. CFD Boundary Conditions			
Lance inlet type	Pressure inlet	Domain outlet type	Pressure outlet
Inlet gauge total pressure	850,000 Pa	Outlet gauge pressure	0 Pa
Inlet initial gauge pressure	2027 Pa	Primary/Secondary phase	Oxygen/Molten steel
Inlet turbulence intensity	5%	Inlet turbulent viscosity ratio	10
Wall boundary condition	No-slip stationary	Near-wall treatment	Standard wall function
3. Physical Properties			
Oxygen (O_2_)			

**Table 3:** Grid Convergence Index (GCI) calculation parameters based on axial velocity at Z = 0.5 m.

Parameter	Symbol	Value
Grid scale		
Base mesh cells	N1	3.0 × 10^5^
Medium mesh cells	N2	5.0 × 10^5^
Fine mesh cells	N3	7.0 × 10^5^
Target Variable (Velocity at Z = 0.5 m)		
Base mesh velocity (m/s)	V1	465.25
Medium mesh velocity (m/s)	V2	497.46
Fine mesh velocity (m/s)	V3	494.21
GCI Calculation Results		
Apparent order of accuracy	p	2.0
Relative error (Medium-Fine)	$e_{a}^{21}$	0.66%
Grid Convergence Index	$GCI_{medium,fine}$	3.27%

**Table 4:** Comparison between CFD simulation and isentropic calculation for the central nozzle.

Parameter	Isentropic Theoretical Value	CFD Simulated Value	Error (%)
Exit Mach number	2.1	2.02	3.97%
Mass flow rate (kg/s)	3.27	3.34	2.10%

**Table 5:** Variation in impact depth over time for different oxygen lance configurations.

	t = 1.1 s	1.2 s	1.3 s	1.4 s	1.5 s	1.6 s	1.7 s	1.8 s	1.9 s	2.0 s
1#	0.188	0.185	0.232	0.264	0.211	0.265	0.247	0.23	0.229	0.23
2#	0.36	0.415	0.251	0.353	0.218	0.251	0.244	0.337	0.306	0.22
3#	0.32	0.306	0.304	0.292	0.247	0.244	0.277	0.339	0.383	0.372
4#	0.155	0.142	0.15	0.235	0.183	0.181	0.179	0.177	0.346	0.183
5#	0.221	0.201	0.238	0.256	0.185	0.174	0.219	0.276	0.297	0.225
6#	0.204	0.205	0.22	0.219	0.259	0.197	0.219	0.24	0.265	0.314
7#	0.251	0.248	0.276	0.251	0.214	0.231	0.225	0.291	0.189	0.255

**Table 6:** Variation in impact radius over time for different oxygen lance configurations.

	t = 1.1 s	1.2 s	1.3 s	1.4 s	1.5 s	1.6 s	1.7 s	1.8 s	1.9 s	2.0 s
1#	0.803	0.579	0.622	0.644	0.702	0.749	0.787	0.8	0.657	0.699
2#	0.699	0.715	0.771	0.881	0.66	0.697	0.726	0.751	0.765	0.794
3#	0.744	0.779	0.66	0.71	0.578	0.626	0.687	0.726	0.752	0.596
4#	0.756	1.006	0.843	0.861	0.762	0.76	0.689	0.698	0.709	0.726
5#	0.809	0.864	1.081	0.77	0.79	0.8	0.744	0.756	0.759	0.768
6#	0.769	0.776	0.793	0.839	1.109	0.776	0.775	0.775	0.766	0.718
7#	0.785	0.799	1.197	1.137	0.776	0.755	0.736	0.718	0.752	0.804

**Table 7:** Sensitivity analysis of macroscopic hydrodynamic parameters under ±5% inlet pressure perturbations (Baseline: Case 2#).

Operating Condition	Inlet Total Pressure (Pa)	Average Impact Depth (m)	Relative Change in Depth	Inactive Zone Ratio (%)	Relative Change in Inactive Zone
−5% Decompression	807,500	0.310	4.7%	0.153	5.5%
Baseline (Case 2#)	850,000	0.296	-	0.145	-
+5% Overpressure	892,500	0.273	7.8%	0.136	6.2%

## Data Availability

The original contributions presented in this study are included in the article. Further inquiries can be directed to the corresponding author.
